# Evaluation of classical swine fever E2 (CSF-E2) subunit vaccine efficacy in the prevention of virus transmission and impact of maternal derived antibody interference in field farm applications

**DOI:** 10.1186/s40813-020-00188-6

**Published:** 2021-01-11

**Authors:** Jing-Yuan Chen, Chi-Ming Wu, Zeng-Weng Chen, Chih-Ming Liao, Ming-Chung Deng, Min-Yuan Chia, Chienjin Huang, Maw-Sheng Chien

**Affiliations:** 1 Graduate Institute of Veterinary Pathobiology, College of Veterinary Medicine, National Chung Hsing University, 145 Xingda Road, Taichung, 40227 Taiwan, Republic of China; 2 Animal Technology Laboratories, Agricultural Technology Research Institute, No. 52, Kedong 2nd Rd., Zhunan Township, Miaoli County, 350401 Taiwan, Republic of China; 3 Animal Health Research Institute, Council of Agriculture, 376 Chung-Cheng Road, Tansui, Taipei, 25158 Taiwan, Republic of China; 4 Department of Veterinary Medicine, College of Veterinary Medicine, National Chung Hsing University, 145 Xingda Road, Taichung, 40227 Taiwan, Republic of China; 5 Graduate Institute of Microbiology and Public Health, College of Veterinary Medicine, National Chung Hsing University, 145 Xingda Road, Taichung, 40227 Taiwan, Republic of China

**Keywords:** Classical swine fever, CSF-E2 subunit and live attenuated CSF vaccine, Maternally derived antibody, Saliva monitoring, Viral RNA detection, Vaccination-challenge-cohabitation trial

## Abstract

**Background:**

Classical swine fever (CSF) is one of the most devastating pig diseases that affect the swine industry worldwide. Besides stamping out policy for eradication, immunization with vaccines of live attenuated CSF or the CSF-E2 subunit is an efficacious measure of disease control. However, after decades of efforts, it is still hard to eliminate CSF from endemically affected regions and reemerging areas. Most of previous studies demonstrated the efficacy of different CSF vaccines in laboratories under high containment conditions, which may not represent the practical performance in field farms. The inadequate vaccine efficacy induced by unrestrained factors may lead to chronic or persistent CSF infection in animals that develop a major source for virus shedding among pig populations. In this study, a vaccination-challenge-cohabitation trial on specific-pathogen-free (SPF) pigs and long-term monitoring of conventional sows and their offspring were used to evaluate the efficacy and the impact of maternally derived antibody (MDA) interference on CSF vaccines in farm applications.

**Results:**

The trials demonstrated higher neutralizing antibody (NA) titers with no clinical symptoms and significant pathological changes in the CSF-E2 subunit vaccine immunized group after CSFV challenge. Additionally, none of the sentinel pigs were infected during cohabitation indicating that the CSF-E2 subunit vaccine could provoke adequately acquired immunity to prevent horizontal transmission. In field farm applications, sows immunized with CSF-E2 subunit vaccine revealed an average of higher and consistent antibody level with significant reduction of CSF viral RNA detection via saliva monitoring in contrast to those of live attenuated CSF vaccine immunized sows possessing diverse antibody titer distributions and higher viral loads. Furthermore, early application of the CSF-E2 subunit vaccine in 3-week-old piglets illustrated no MDA interference on primary immunization and could elicit consistent and long-lasting adequate antibody response suggesting the flexibility of CSF-E2 subunit vaccine on vaccination program determination.

**Conclusions:**

The CSF-E2 subunit vaccine demonstrated significant efficacy and no MDA interference for immunization in both pregnant sows and piglets. These advantages provide a novel approach to avoid possible virus shedding in sow population and MDA interference in piglets for control of CSF in field farm applications.

**Supplementary Information:**

The online version contains supplementary material available at 10.1186/s40813-020-00188-6.

## Background

Classical swine fever (CSF, formerly known as hog cholera) is one of the most devastating and transboundary viral diseases of swine worldwide [1]. CSF is caused by the classical swine fever virus (CSFV), an enveloped single-stranded RNA virus of positive polarity from the family *Flaviviridae* and genus *Pestivirus*. The CSFV genome possesses one open reading frame encoding a polyprotein which undergoes transcriptional and post-transcriptional modifications by the host cellular or viral protease to produce structure (Core, E^rns^, E^1^, E^2^) or non-structure (N^pro^, P7, NS2, NS3, NS4A/B, NS5A/B) proteins [2, 3]. The virulence of the CSFV strain has been divided into three levels (low, medium, and high), which are associated with different clinical progression and induced pathological lesions [4–6]. In addition, depending on the variety of CSFV virulence and the triggered inflammatory response in infected pigs, the course of CSF infection can be classified as acute, chronic, and persistent [4, 7, 8]. An acute CSF pattern, which includes high fever, anorexia, conjunctivitis, leukopenia, thrombocytopenia, massive hemorrhage, and enlarged lymph nodes are noticed at the early stage of infection with high-virulent CSFV [7, 9–11]. Death may be observed within 2 to 4 weeks after exposure and the mortality may reach up to 100% [8]. However, pigs infected with medium or low virulent CSFV strains may develop a chronic or persistent course of disease with inapparent clinical signs that are quite difficult to be identified via the clinical diagnosis. In fact, chronic or persistent CSF infected pigs may reside in the farm until transportation for slaughter to market. It is presumed that these infected pigs represent a major source of CSFV and shed virus continuously or intermittently in field farms leading to a risk of high exposure in the healthy population [6, 12].

Vaccination is one of the most effective strategies to control and prevent a CSF outbreak in endemic regions [3, 8, 13]. Several live attenuated CSF vaccines have been developed via a series of passages in virulent CSFV on rabbits, guinea pigs, or adapted cultures in cell lines, and have been used in field farms for decades [3, 13]. It has been demonstrated that a live attenuated CSF vaccine can provide protective efficacy as early as 5 days after vaccination and induce humoral immunity for long-term protection [13–16]. However, several issues, such as stability of vaccine batches, loss of the cold chain during transportation, concurrent or secondary infection with other pathogens during vaccination, and the interference of MDA, may lead to variant vaccination efficacy among areas or countries [6, 17, 18]. However, it is difficult to differentiate infected pigs from vaccinated animals with live attenuated vaccines [19, 20]. Moreover, recent studies have revealed that the immunity induced by live attenuated vaccines may not eradicate the virus in clinical applications due to the circulation of medium and low virulence strains in field farms [5, 6]. An incomplete immune response induced by vaccination has been considered a positive selection acting for viral evolution [21, 22]. Therefore, despite the effective minimization of disease outbreak with the use of live attenuated CSF vaccines, the biosecurity and the capability of virus eradication in field farm applications needs to be evaluated.

Several subunit marker vaccines based on the envelope glycoprotein E2 (CSF-E2), which is the major antigen to elicit neutralizing antibodies against CSFV have been studied and reviewed [19, 23–26]. Previous studies mainly performed the vaccination-challenge model under high containment conditions to evaluate the efficacy of CSF-E2 subunit marker vaccines [27–29]. Lots of prototype vaccines have been studied in laboratories; however, only a few of them have been authorized for field farm applications, and studies that have focused on field farm applications, especially in sows and offspring, are rare. Accordingly, we have conducted a vaccination-challenge and cohabitation trial by using SPF pigs as sentinel animals to evaluate the efficacy of the CSF-E2 subunit vaccine against high-virulent CSFV challenge and its ability for reducing viral horizontal transmission. In addition, an assessment of the impact of high MDA levels on CSF-E2 subunit vaccine or live attenuated CSF vaccines was conducted to evaluate vaccine efficacy and vaccination strategies in the field farm.

## Methods and methods

### Animals and farm selection

Ten six-week-old SPF pigs were purchased from Animal Technology Laboratories, Agricultural Technology Research Institute, Miaoli, Taiwan for vaccination-challenge experiments. A farrow-to-finish continuous flow production pig farm located in Taichung, Taiwan was selected for this study. There were 150 Landrace-Yorkshire sows and 2,000 Landrace-Yorkshire-Duroc pig in the farm. The mean farrowing number per sow was 11 and the mean weaning number per litter was 10. The mean raised piglets per sow per year was 19.8. The live attenuated CSF vaccine (Lapinized Philippines Coronel strain, LPC vaccine) was used routinely on the farm to prevent the outbreak of CSF. Gilts and nursery pigs in the farm received two doses of LPC vaccine at 6 and 9 weeks of age, respectively, based on the prime-boost vaccination program. Sows were routinely immunized with one dose of LPC vaccine before insemination. Besides the LPC vaccine, sows were routinely immunized with porcine circovirus type 2 (PCV2), pseudorabies and atrophic rhinitis vaccines, and piglets were immunized with PCV2, atrophic rhinitis, enzootic pneumonia, and pseudorabies vaccines to prevent diseases. There was no CSF outbreak record noted in this conventional farm. All animals in the study were fed ad libitum and raised in a high containment animal biosecurity level II (ABSL-2) unit (SPF pigs) or the original field farm (conventional pigs). All animal trials and experimental procedures were reviewed and approved by the Institutional Animal Care and Use Committee (IACUC) of National Chung Hsing University under IACUC approval number 98–64 and 105–005.

### CSF vaccines

**Fig. 1:**
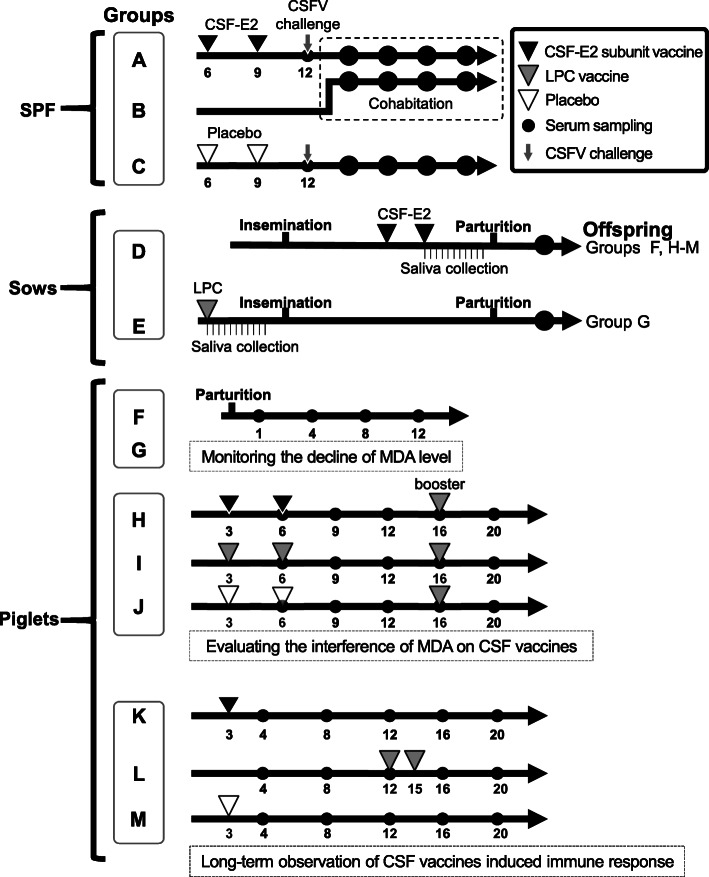
Schema of the experimental design in this study

The CSF-E2 subunit vaccine (Bayovac® CSF-E2 vaccine, Bayer Taiwan Co., Ltd.) and the LPC vaccine (Frozen dried lapinized hog cholera vaccine, Animal Health Research Institute, Taiwan) were used in this study. For immunization with a single dose (2 mL) of each vaccine, pigs were injected at the neck behind the ear intramuscularly. To evaluate the efficacy of the CSF-E2 subunit vaccine, a vaccination-challenge trail was performed on SPF pigs (trial I). Three other trials (trials II–IV), which included the surveillance of sows and piglets, were designed and performed to evaluate the application of the CSF-E2 subunit vaccine in field farms (Fig. 1 and Table 1).

**Table 1:** Pig groups and experiment schedules in the study

Trials	Categories	Groups	Animal number	Vaccines^a^	Vaccination program	Challenge^b^/booster^c^	Serum sampling
I	SPF	A	4	CSF-E2	6 and 9 weeks old	CSFV ALD strain	0, 4, 7, 12, 17, 25 DPC
		B	4	–	–	Cohabitation at 4 DPC	4, 7, 12, 17 DPC
		C	2	Placebo	6 and 9 weeks old	CSFV ALD strain	0, 4, 7 DPC
II	Sow	D	25	CSF-E2	−5 and −3 weeks before parturition	–	3 days after parturition
		E	35	LPC	Once before insemination	–	3 days after parturition
	Piglet	F^d^	20	–	–	–	1, 4, 8,12 weeks old
		G^e^	20	–	–	–	1, 4, 8,12 weeks old
III	Piglet^d^	H	6	CSF-E2	3 and 6 weeks old	LPC vaccine	6, 9, 12, 16, 20 weeks old
		I	6	LPC	3 and 6 weeks old	LPC vaccine	6, 9, 12, 16, 20 weeks old
		J	6	Placebo	3 and 6 weeks old	LPC vaccine	6, 9, 12, 16, 20 weeks old
IV	Piglet^d^	K	10	CSF-E2	3 weeks old	–	4, 8, 12, 16, 20 weeks old
		L	10	LPC	12 and 15 weeks old	–	4, 8, 12, 16, 20 weeks old
		M	10	Placebo	3 weeks old	–	4, 8, 12, 16, 20 weeks old

^a^CSF-E2 vaccine, baculovirus-expressed CSF-E2 subunit protein emulsified with water-in-oil (W/O) adjuvant; LPC vaccine, live attenuated CSF vaccine; Placebo, 0.9% saline emulsified with the W/O adjuvant

^b^Groups A and C were challenged with 1 × 10^5^ TCID_50_ CSFV (ALD strain) intramuscularly on the neck behind the ear at 12 weeks old

^c^Group H-J pigs were immunized with one dose of LPC vaccine at 16 weeks old as a booster

^d^Offspring from 5 sows in group D with CSF-specific antibody blocking percentage over 85% were assigned to group F (4 piglets per litter)

^e^Offspring from 5 sows in group E with CSF-specific antibody blocking percentage over 85% were assigned to group G (4 piglets per litter)

### Animal trial design

In trial I, the efficacy of the CSF-E2 subunit vaccine on reducing CSFV horizontal transmission was evaluated. Ten six-week-old SPF pigs were used in the vaccination-challenge trial and randomly allotted to three groups. Group A pigs (*n* = 4) were vaccinated with the CSF-E2 subunit vaccine at 6 and 9 weeks of age. Three weeks after the vaccination, pigs were challenged with 1 × 10^5^ 50% tissue culture infective dose (TCID_50_) of high virulence CSFV ALD strain via intramuscular injection at 12 weeks of age. Four days after the CSFV challenge, the sentinel pigs (group B, *n* = 4) were transferred to cohabitate with group A to assess horizontal viral transmission. Control group C pigs (*n* = 2) vaccinated with placebo at 6 and 9 weeks of age were also challenged with 1 × 10^5^ TCID_50_ of the CSFV ALD strain at the same age as group A. The pigs in group C were euthanized at 7 days post-challenge (DPC) due to severe clinical symptoms. The surviving pigs in groups A and B were euthanized for pathological examination at 25 DPC. The central nervous system (cerebrum and cerebellum), spleen, tonsil, lymph nodes and kidney were collected on necropsy and fixed in 10% neutral formalin for microscopic examination. The paraffin-embedded tissue sections were examined and blind scored by three trained pathologists according to the histopathological score system described by Malswamkima et al., in previous study (0–3 scale: normal-0, mild-1, moderate-2, severe-3) [30]. The ethylenediaminetetraacetic acid (EDTA) anticoagulant blood and serum samples were collected before the CSFV challenge (day = 0) and at 4, 7, 12, 17, and 25 DPC. Since leukopenia and thrombocytopenia are characteristic findings of acute CSFV infection, the EDTA-anticoagulant blood was subjected to a complete blood count by using ProCyte Dx™ (IDEXX Laboratories, Inc., Westbrook, ME, USA). A leukocyte count below 11 × 10^3^ cells/μL and a platelet count below 211 × 10^3^ cells/μL were considered as leukopenia and thrombocytopenia [27, 31, 32]. Serum samples were used to analyze CSFV-specific NA level and viremia levels.

In trial II, 60 sows from the conventional pig farm with a routine LPC vaccination before insemination were randomly divided into two groups to analyze the immune response induced by the CSF-E2 subunit vaccine (group D, *n* = 25) and LPC vaccine (group E, *n* = 35). Sows in group D were immunized with two doses of CSF-E2 subunit vaccine at 3 and 5 weeks before parturition, whereas sows in group E were immunized with one dose of LPC vaccine before insemination according to the original vaccination program in the conventional pig farm. The saliva samples were collected using cotton ropes from five sows (total ten sows) in each group at the day of vaccination (day 0) and in 3-day intervals for 1 month (day 30) [33]. Serum samples of sows in group D and group E were collected 3 days after parturition for analysis of CSF-specific antibody level as the prospective MDA levels (Fig. 1). Furthermore, the offspring from each group were monitored to profile the decline of MDA. Offspring from 5 sows in group D with CSF-specific antibody blocking percentage over 85% were assigned to group F (*n* = 20, 4 piglets per litter), and offspring from 5 sows in group E with CSF-specific antibody blocking percentage over 85% were assigned to group G (*n* = 20, 4 piglets per litter). Piglets in groups F and G were non-vaccinated and serum samples were collected at 1, 4, 8, and 12 weeks of age to monitor acquired immunity and the decline of the MDA level.

In trial III, 18 piglets from group D with high CSF-specific antibody level (mean blocking percentage 88.89% ± 0.94% at 3 weeks of age) were randomly assigned to one of three groups and immunized with CSF-E2 subunit vaccine (group H, *n* = 6), LPC vaccine (group I, *n* = 6), or placebo (group J, *n* = 6) at 3 and 6 weeks of age to evaluate the potential interference of MDA on the vaccine-induced immune response (Table 1). Serum samples were collected at 6, 9, 12, 16, and 20 weeks of age and the CSF-specific antibody level was monitored. Due to consideration of biosecurity, all pigs in trial III were boosted with live attenuated CSFV (LPC vaccine) at 16 weeks of age to mimic the possible contamination from chronic or persistent CSFV infection in conventional pig population. Four weeks after the boost, the CSF-specific antibody level was screened to evaluate and clarify the interference of high-level MDA on the CSF vaccine-induced immune response.

In trial IV, the CSF vaccine-induced immune response in field farm applications was performed with long-term observation from weaning to the finishing stage. Thirty piglets from group D were randomly assigned to one of three groups (Table 1). Group K (*n* = 10) was immunized with one dose of the CSF-E2 subunit vaccine at 3 weeks of age, and group L (*n* = 10) was immunized with two doses of the LPC vaccine at 12 and 15 weeks of age. Group M (*n* = 10) were injected with a placebo once at 3 weeks of age and used as the control group. Serum samples were collected at 4, 8, 12, 16, and 20 weeks of age and the CSF-specific antibody level was monitored.

### Detection of CSFV RNA by real-time PCR

Serum RNA samples were extracted using a NucleoSpin® RNA kit (740,955.50, Macherey-Nagel GmBH & Co. KG, Duren, Germany) according to the manufacturer’s instruction and saliva RNA samples were extracted using RNAzol®RT (R4533, Sigma-Aldrich, St. Louis, MO, USA). All RNA samples were reverse transcribed with an iScript cDNA synthesis kit (1,708,891, Bio-Rad, Hercules, CA, USA) according to the manufacturer’s procedures. The real-time PCR assay specific for CSFV 5’UTR sequence utilized in this study has been described and validated in the study by Hoffmann et al., 2005 [34]. The real-time PCR was performed using a LightCycler®480 instrument (Roche diagnostic GmBH, Mannheim, Germany) and the crossing point (Cp) value of each reaction was calculated using LightCycler®480 software version 1.5 (Roche Life Science). The quantification data were Log_10_ transformed for analysis.

### Detection of CSF-specific antibody in pig serum samples

The CSFV-specific NA level against the CSFV (LPC strain) was determined using a fluorescent antibody virus neutralization assay according to the diagnostic manual of OIE (World Organization for Animal Health) [35]. The NA level was Log_2_ transformed analysis. According to Terpstra et al. (1988), pigs with NA level less than 12.5 cannot prevent disease and death, pigs with NA level between 12.5 and 30 may survive from virus challenge but insufficient to avoid excretion of virus, whereas the NA level greater than 1:32 was considered adequate to protect individual pig and prevent virus transmission in the population [13, 36]. The serum CSF-specific antibody level was also analyzed with a competitive ELISA kit, the IDEXX CSF Ab test kit (IDEXX Laboratories Inc., Liebefeld, Switzerland) according to the manufacturer’s instructions. The results were expressed as the blocking percentage and a blocking percentage greater than 40% was considered positive and recognized adequate to prevent CSFV infection (Additional file 1: Figure S1).

### Statistical analysis

The positive percentage of saliva CSF RNA was calculated and Pearson’s chi-square test with Yate’s continuity correction was used for statistical analysis. The serum antibody level was expressed as the mean ± standard error of the mean. The coefficient of variation (CV) value of antibody level was calculated and expressed as a percentage to represent the variation of antibody level in each group. Welch’s two-sample t-test was used to evaluate the antibody levels between groups in trial II. A one-way analysis of variance followed by a Tukey post hoc test was used to evaluate the antibody level between groups in trials III and IV. Data analysis was performed using R software version 3.6.1 (The R Foundation, Vienna, Austria), and differences were considered statistically significant for a *p* value less than 0.05.

## Results

### CSF-E2 subunit vaccine could protect pigs against CSFV challenge and completely prevent horizontal transmission

**Fig. 2:**
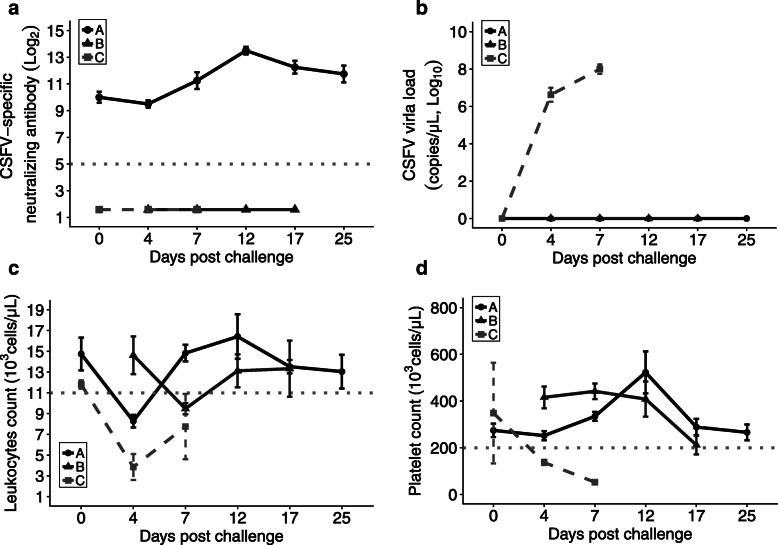
The CSF-E2 subunit vaccine could prevent CSFV horizontal transmission on a vaccination–challenge animal trial. SPF pigs were immunized with CSF-E2 subunit vaccine (group A, circle and black line) or placebo (group C, square and light gray dashed line) and challenged with CSFV ALD strain (1 × 10^5^ TCID_50_) after two vaccinations. Groups A and C were raised in two isolated ABSL-2 units. The sentinel SPF pigs (group B, triangle and gray line) were cohabited with group A at 4 days after the virus challenge to monitor the transmission of CSFV. Pigs in group C were sacrificed 7 days after the virus challenge due to severe clinical symptoms. **a** The CSFV-specific neutralizing antibody level was analyzed after the CSFV challenge. The gray dotted line indicated the protective neutralizing antibody level (1:32). **b** The viremia level was monitored after the virus challenge to identify the infection of CSFV. **c** and **d** The number of leukocytes and platelets were counted after virus challenge to monitor CSFV infection-induced leukopenia (< 11,000 cells/μL, gray dotted line) and thrombocytopenia (< 211,000 cells/μL, gray dotted line)

The efficacy of the CSF-E2 subunit vaccine was demonstrated in the vaccination-challenge trial by using SPF pigs. Pigs in group A were immunized with CSF-E2 subunit vaccine and group C pigs were immunized with placebo at 6 and 9 weeks of age, respectively. After vaccination, pigs of groups A and C were challenged with the high virulence CSFV ALD strain at 12 weeks of age (0 DPC). The pigs in group B were sentinel animals that were transferred to group A to cohabitate at 4 DPC to assess the transmission of CSFV. After the virus challenge, the group C pigs showed cyanosis on the tip of ears and legs and several characteristic pathological findings were noted on necropsy at 7 DPC (Additional file 1: Figure S2 and Figure S3). The microscopic examination result showed lower histopathological score on group A and cohabitated sentinel group B than group C, indicating the protective efficacy of the CSF-E2 subunit vaccine (Table 2). Pigs in group A (10.00 ± 0.41 log_2_, 0 DPC) showed significantly higher CSFV-specific NA level after two vaccinations than group C (1.59 ± 0.00 log_2_, 0 DPC). After the challenge by the CSFV ALD strain, group A showed significant conversion to the NA level between 4 (9.50 ± 0.29 log_2_) and 12 (13.50 ± 0.29 log_2_) DPC, whereas no antibody response was noted in groups B and C (Fig. 2a). The viral load was remarkably increased in group C between 0 (negative) and 7 (8.00 ± 0.25 log_10_) DPC representing the infection of CSFV. In contrast, there was no detectable viremia level in pigs of group A (CSF-E2 subunit vaccine immunized pigs) before virus challenge (0 DPC) and throughout the experiment period (25 DPC), suggesting the CSF-E2 subunit vaccine immunized pigs showed no viremia detecting after CSFV challenge. More importantly, cohabitation sentinel pigs showed no clinical signs of infection with no viremia detecting during entire experiment period, indicating the CSF-E2 immunized pigs could prevent horizontal transmission after CSFV challenge (Fig. 2b). The leukocyte count was decreased after virus challenge in groups A (8.28 ± 0.61 × 10^3^ cells/μL) and C (3.85 ± 1.25 × 10^3^ cells/μL) at 4 DPC indicating the impact of CSFV infection on pigs (Fig. 2c). The leukocyte count in group A pigs rapidly recovered at 7 DPC showing a vaccination-induced protective immune response, whereas pigs in group C remained status of leukopenia. Moreover, the number of platelets decreased steeply after the virus challenge in group C (52.50 ± 3.50 × 10^3^ cells/μL) at 4 DPC which might be associated with the hemorrhage lesions noted on the kidney and ileocecal valve (Fig. 2d & Additional file 1).

**Table 2:** Histopathological score of groups A, B, and C

		Groups	A				B				C	
Organs	Criteria^a^	Pigs	A1	A2	A3	A4	B1	B2	B3	B4	C5	C6
Central nervous system	Cuffing		1	0	1	0	0	0	0	0	3	3
	Endothelium hypertrophy/ hyperplasia		1	1	1	1	1	1	1	1	3	3
	Gliosis		1	1	1	0	0	0	0	1	3	3
Spleen	Lymphoid necrosis		0	0	0	0	0	0	0	0	0	0
	Lymphoid depletion		0	0	1	0	0	1	0	1	2	2
Tonsil	Lymphoid necrosis		1	1	1	1	1	1	1	1	3	1
	Lymphoid depletion		0	0	0	0	0	0	0	0	0	1
Lymph nodes	Lymphoid depletion		1	1	1	1	0	0	0	0	2	2
	Lymphoid necrosis		0	0	0	0	0	0	0	0	1	0
Kidney	Interstitial nephritis		0	0	0	1	0	0	0	0	2	0
	Total score		5	4	6	4	2	3	2	4	19	15

^a^The histopathological score system used in the study was described by Malswamkima et al., in previous study (0–3 scale: normal-0, mild-1, moderate-2, severe-3). The tissues including central nervous system, spleen, tonsil, lymph nodes and kidney were blind scored by three trained pathologists

### CSF-E2 subunit vaccine induced a stronger and more consistent immune response in sows to provide long-lasting MDA

**Fig. 3:**
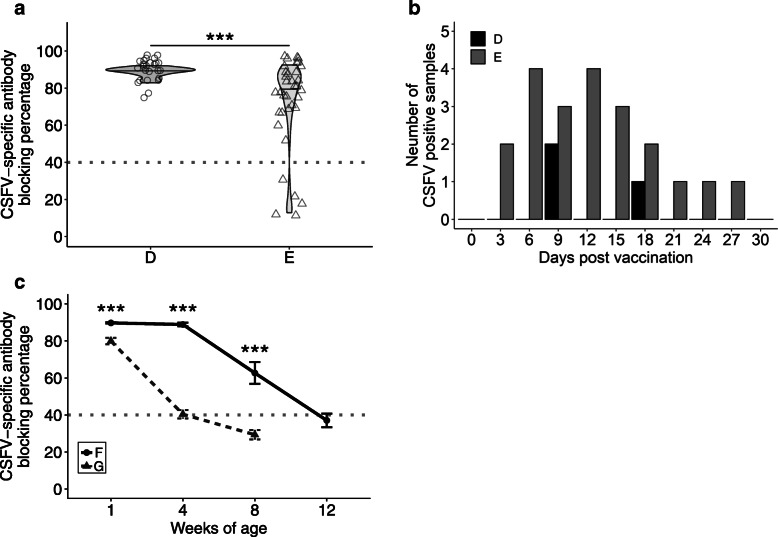
Evaluation of vaccine induced immune response on sows and the passive immunity of piglets. Sows were immunized with two doses of CSF-E2 vaccine at 3 and 5 weeks before parturition (group D, *n* = 25) or one dose of LPC vaccine before insemination (group E, *n* = 35). **a** The CSF-specific antibody level was detected using an IDEXX CSFV Ab test kit to monitor vaccine induced immune response. The gray dotted line indicates a positive antibody response (blocking percentage > 40%). **b** The saliva CSFV RNA was assessed for 1 month after vaccination using real-time PCR. Pearson’s chi-square test with Yate’s continuity correction showed a significant difference between the CSFV positive count of groups D and E (*p* < 0.001). **c** The piglet serum samples randomly selected from CSF-E2 vaccinated sows (group F, *n* = 20) and LPC vaccinated sows (group G, *n* = 20) were analyzed by using the IDEXX CSFV Ab test kit. The mean blocking percentage of each group was calculated and Welch’s two-sample t-test was performed to evaluate the statistical difference between groups. *** *p* < 0.001

To evaluate a feasible program of CSFV vaccination, sows in group D were immunized with CSF-E2 subunit vaccine at 3 and 5 weeks before parturition, whereas sows in group E remained in the ordinary LPC vaccination program of the conventional pig farm. There were no reproductive problems and any adverse effects associated with CSF vaccines immunization noted in group D and group E. At 3 days post-parturition, sows in group D (88.75% ± 2.54%) showed significantly higher antibody levels than group E (71.66 ± 24.28; *p* < 0.05) (Fig. 3a). The CV in group D (2.86%) was lower than in group E (33.88%), indicating more consistent antibody level distribution in CSF-E2 subunit vaccine immunized sows. Moreover, the detection of saliva CSFV RNA in sows may reveal the possible vertical transmission of virus in the field farm. After immunization with the CSF-E2 subunit vaccine, there was a significantly lower CSFV RNA positive ratio in the sows of group D (6.00%) than those of group E (42.00%; *p* < 0.001) (Fig. 3b).

The passive immunity from colostrum is crucial for piglets to prevent infection by pathogens during the suckling to the weaning phase and the passive immunity may decline gradually after weaning. Monitoring the dynamics of MDA has been widely used for making the vaccination program of live attenuated CSF vaccine in field farms. Piglets in group F (offspring from CSF-E2 subunit vaccine immunized sows in group D) have a significantly higher CSF-specific antibody level than piglets in group G (offspring from LPC vaccine immunized sows in group E) at 1 (89.73% ± 0.33% versus 79.92% ± 1.76%), 4 (88.89 ± 0.94% versus 40.41% ± 2.31%), and 8 (62.68% ± 5.88% versus 29.33% ± 2.57%) weeks of age (Fig. 3c). The MDA of group F declined gradually to a low level of 37.08% ± 3.70% at 12 weeks of age (Fig. 3c).

### The high level of MDA did not interfere with CSF-E2 vaccine induced immune response

**Fig. 4:**
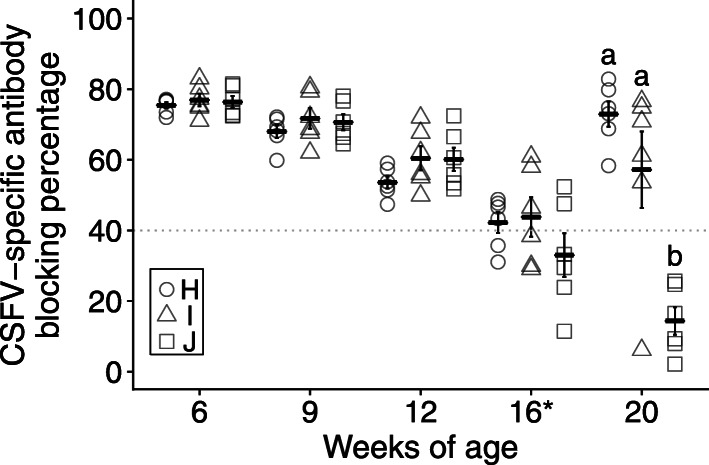
The interference of maternally derived antibodies on CSFV vaccines. Piglets with high maternal derived antibody were immunized with two doses of CSF-E2 vaccine (group H, *n* = 6), LPC vaccine (group I, *n* = 6), or placebo (group J, *n* = 6) at 3 and 6 weeks of age and received a booster immunization with one dose of LPC vaccine at 16 weeks of age (*). The dynamics of the CSF-specific antibody level were monitored by using an IDEXX CSFV Ab blocking ELISA test kit and the gray dotted line indicated adequate antibody level (blocking percentage > 40%). Different letters indicate statistically significant difference (*p* < 0.001)

In trial III, the interference of MDA on CSF vaccine induced immune response was estimated. The piglets from group D with a high MDA level (mean blocking percentage 88.94% ± 0.94%) were randomly divided into three groups and immunized with two doses of CSF-E2 subunit vaccine (group H), LPC vaccine (group I), or placebo (group J) at 3 and 6 weeks of age. All pigs in trial III were boosted with one dose of LPC vaccine at 16 weeks of age. The dynamic of CSF-specific antibody levels was monitored to compare memory immunity induced by different CSFV vaccines. After the LPC booster vaccine, pigs in group H (72.94% ± 3.56%, CV 11.95%) and group I (57.21% ± 10.82%, CV 40.31%) had a significantly higher CSF-specific antibody level than group J (14.38% ± 3.89%, CV 66.29%) at 20 weeks of age (Fig. 4). Although groups H and I had an adequate mean value of antibody level and no statistical difference was noted at 20 weeks of age, group H pigs had rapidly seroconverted after the boost, whereas one of six pigs in group I showed no response to the LPC booster vaccine suggesting that MDA has an impact on the LPC vaccine-induced immune response.

### Application of the CSF-E2 subunit vaccine in field farm applications with long-term observation from weaning to finishing

**Fig. 5:**
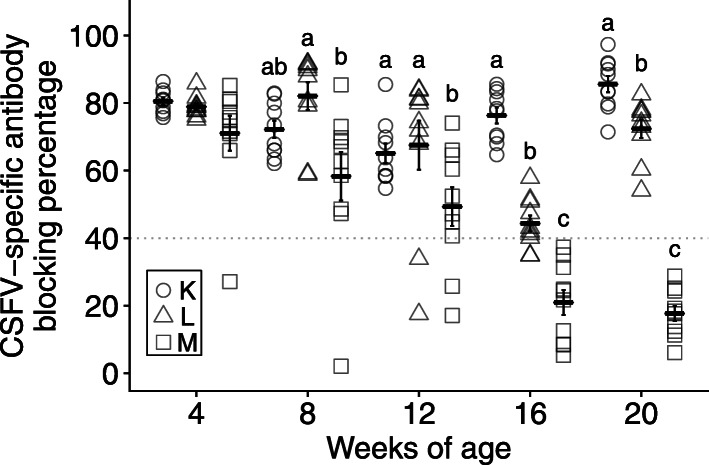
Application of CSFV vaccines in a field farm with long-term observation. Pigs were divided into three groups and immunized with one dose of CSF-E2 vaccine (group K, *n* = 10) or placebo (group M, *n* = 10) at 4 weeks of age or two doses of LPC vaccine (group L, *n* = 10) at 12 and 16 weeks of age. The serum samples were collected from weaning to finishing in four-week intervals and analyzed using an IDEXX CSFV Ab blocking ELISA test kit. The gray dotted line represents the adequate antibody level (blocking percentage > 40%) and different letters indicate a statistically significant difference (*p* < 0.05)

In trial IV, the immune response of the CSF-E2 subunit vaccine or the combination with the LPC vaccine was evaluated. Thirty piglets from the CSF-E2 subunit vaccine immunized sows (group D) were randomly divided into three groups (ten piglets in each group) and immunized with one dose of CSF-E2 subunit vaccine at 4 weeks of age (group K), two doses of LPC vaccine at 12 and 16 weeks of age (group L), and placebo once at 4 weeks of age (group M), respectively. At 4 weeks of age, there was no statistical difference in the CSF-specific antibody level between the three groups (K: 80.50% ± 1.06%; L: 78.98% ± 0.95%; M: 71.03% ± 5.19%), an adequate MDA level was displayed in all piglets. The antibody level of group M declined during the experimental period and, compared with groups K and L, a significantly lower antibody level was noted at 12 (K: 65.11% ± 2.95%; L: 65.52% ± 7.27%; M: 49.31% ± 5.73%), 16 (K: 76.35% ± 2.37%; L: 44.34% ± 2.35%; M: 20.96% ± 3.69%), and 20 (K: 85.60% ± 2.43%; L: 72.39% ± 2.77%; M: 17.70% ± 2.22%) weeks of age. Group K had a significantly higher antibody level than group L at 16 and 20 weeks of age. During the observation period, all pigs in group K had a high and consistent antibody level suggesting that the protective efficacy of the CSF-E2 subunit vaccine was adequate. However, a few pigs in group L (2 of 10) had an insufficient antibody level at 12 and 16 weeks of age and could be under the risk of field virus infection (Fig. 5).

## Discussion

CSF is one of the most historic and devastating pig diseases that affect the swine industry worldwide [9, 37]. The impact of the CSF outbreak may bring tremendous socio-economical losses among different levels of pig production from backyard small scale to industrial-scale farming. Once a disease outbreak occurs, several long-lasting processes are needed for endemic countries to be recognized as CSF-free by the World Organization for Animal Health (OIE) and return to the world trade market [38]. In many endemic regions, immunization with live attenuated CSF vaccine is crucial to prevent economic losses that are addressed in the context of a national control program [20, 38]. In 2015, Japan was officially announced by OIE to be CSF-free and added to the list of countries with CSF-free status after conducting a successful 10-year eradication program; however, several sporadic reemerging incursions transmitted from wild boar to the domestic population have been reported recently [39–41]. In addition, CSF reemerged accidentally in the Jeju island of South Korea after the unintentional vaccination of live attenuated CSF vaccine (LOM strain) on naïve pigs in 2014 [42–44]. The alignment of whole-genome sequences of field virulent CSFV strains in the Jeju island (Jeju LOM strain) and the LOM vaccine strain revealed high identity on nucleotide (98.7–99.0%) and amino acid sequence (98.9–99.2%) [44]. Inoculation trials in SPF pigs and pregnant sows indicated that the CSFV Jeju LOM strain had the most characteristics of the LOM vaccine strains and may cause persistent infection in fetuses [42]. Hence, the safety of live attenuated CSF vaccines should be considered more in field farm applications. This coincidence confirms CSF is indeed a highly contagious and fastidious infectious disease, which resides long-term in farms and fields.

Live attenuated CSF vaccines are effective in reducing economic losses and can prevent pigs from severe clinical symptoms or death, nevertheless, its efficacy may be influenced by several factors [18, 24, 38]. Incomplete vaccine efficacy may lead to virus escape from immunized pigs and act as a positive selection pressure to accelerate virus evolution [21, 22, 44–46]. Other major drawbacks, including variant vaccination protocols in piglets due to deviation of MDA in sows, adverse effects in weak piglets, and interference by concurrent infection of PRRSV (porcine reproductive and respiratory virus), PCV2, and/or bacterial pathogens during immunization, all greatly impact vaccine efficacy in pig farms. Therefore, significant difficulties may occur when the live attenuated CSF vaccine is used for the purpose of virus elimination in endemic regions.

The efficacy of different CSF-E2 subunit protein-based non-infectious marker vaccines has been demonstrated in previous studies [27, 29, 47, 48]. However, incomplete efficacy of a previously authorized subunit marker vaccine (Porcilis® Pesti, Intervet International B.V.) resulting in vertical transmission in a contact-infection gilt experiment has been reported [48]. Nevertheless, the mean NA level of vaccinated gilts before the contact-infection examination was less than the mean of 4 log_2_, which was not sufficient to prevent virus transmission in the pig population [13, 36, 48]. In our study, the CSF-E2 subunit vaccine containing a higher quantity and purity of antigen emulated with designated adjuvant formulation had comprehensive protective efficacy in preventing virus transmission. In trial I, pigs immunized with CSF-E2 subunit vaccine revealed adequate NA level at 10 log_2_ after two doses of vaccination and showed no clinical symptoms, free of viremia detection and pathological changes after 1 × 10^5^ TCID_50_ high-virulent CSFV challenge, indicating the protective efficacy induced by the CSF-E2 subunit vaccine. Based on clinical pathology examinations, a decrease in the concentration of leukocytes was noted in all CSFV challenged pigs at 4 DPC, but only immunized pigs recovered promptly at 7 DPC suggesting that the CSF-E2 subunit vaccine could provoke faster protective immunity. The CSFV challenge had no impact on platelet activity in CSF-E2 subunit vaccine immunized pigs, however, severe thrombocytopenia was noticed in the pigs of the control group (Fig. 2). The thrombocytopenia in non-vaccinated and challenged pigs might be linked to typical turkey-egg kidney hemorrhage or infarct lesions on the kidney, spleen, and ileocecal valve representing acute CSFV infection. Moreover, the screening of viremia and NA level of the sentinel pigs showed no horizontal transmission during cohabitation with vaccinated pigs after challenge and throughout the experimental period. These results demonstrate that the protective efficacy of the CSF-E2 subunit vaccine could completely prevent virus transmission in vaccination-challenge-cohabitation pigs.

To achieve the goal of CSF eradication in the endemic pig farm, an appropriate sequential vaccination procedure should include a safe and efficacious vaccine and flexible immunization programs among populations of sows, piglets, and grower to finisher as well as good biosecurity control measures [24, 38]. In trial II, high safety and good efficacy were obtained with the application of the CSF-E2 subunit vaccine on pregnant sows in field farms. Sows were immunized at 3 and 5 weeks before parturition showed a higher and more consistent antibody level than sows received live attenuated CSF vaccine immunization before insemination (Fig. 3). In addition, according to the screening of CSFV RNA in saliva, the CSF-E2 subunit vaccine immunized sows significantly reduced the ratio of viral RNA, whereas sows immunized with live attenuated CSF vaccine revealed an enormously positive ratio of viral RNA. Since sows make close contact with the fetus at the nursery stage, reducing the viral secretion from sows may minimize the risk of vertical and horizontal transmission to piglets. In a previous study, the viability of piglets from CSFV antibody free sows immunized with live attenuated CSF vaccine at the middle stage of pregnancy (55 days of gestation) was only 34.9%. Although there was no pathological change noted on the necropsy, CSFV RNA was detected in the organs of vaccinated sows and their litters [49]. Since the CSF-E2 subunit vaccine antigen is based on non-infectious recombinant protein, it can be used at any stage of pregnancy to elicit a higher and more consistent immune response. In other words, the sows that possessed a higher antibody level before parturition may provide offspring with a more sufficient passive immunity against infection. The offspring from CSF-E2 subunit vaccine immunized sows in trial II showed significantly higher and long-lasting MDA than those offspring from live attenuated CSF vaccine immunized sows. The higher MDA is vital and essential for protecting piglets from possible early infection before primary vaccination. However, it is demonstrated that high MDA during immunization will impair live attenuated CSF vaccine efficacy; therefore, the vaccination schedule for piglets should be estimated cautiously according to MDA decline [24, 50]. For safety concerns, the application of live attenuated CSF vaccine in sows is required only before insemination to avoid vertical transmission. For this reason, a fluctuation of antibody titers would be noticed among individual sows or gilts. To put it another way, each batch of offspring derived from different sows or gilts would display more variation in passive antibody titer during the nursery to the weaning stage. This will induce prodigious difficulty in scheduling an accurate program for piglet primary vaccination in an endemic farm. In trial II, the MDA level profile showed that sows immunized with CSF-E2 subunit vaccine can provide a consistent and adequate passive immunity to piglets until 12 weeks of age, while piglets from the LPC vaccine immunized sows had insufficient passive immunity and declined quickly between 4 and 8 weeks of age. The unique characteristics of CSF-E2 subunit vaccine from the LPC vaccine allowed immunized pregnant sows to transfer adequate passive immunity in piglets until 12 weeks of age. This provides evidence of the benefits of avoiding CSF primary vaccination during the critical 4 to 8 weeks, which is also the high peak outbreak of PRRSV, PCV2, and/or bacterial pathogens concurrent infections in field farms [17, 18, 51]. It is believed that late CSF immunization in the weaning stage reduces numerous stress factors and MDA interference, and avoiding interference from concurrent infections may offer a more comprehensive and satisfactory humoral immunity in vaccinated pigs.

In trial II, sows immunized with CSF-E2 subunit vaccine were demonstrated to prevent the risk of horizontal or vertical transmission by significantly reducing virus excretion and providing more consistent and long-lasting MDA to their offspring during weaning to nursery stages. For the further evaluation of MDA interference on CSF vaccine application, piglet trials were designed to compare the provoked humoral immune response when piglets immunized with CSF-E2 subunit vaccine or live attenuated CSF vaccine under high levels of MDA interference. Since the MDA levels of offspring from LPC vaccine immunized sows revealed more fluctuant with average lower and fast declining than those offspring from CSF-E2 subunit vaccine immunized sows, piglets from CSF-E2 immunized sows with high CSF-specific antibody levels of blocking percentage (mean blocking percentage 88.94% ± 0.94%, equivalent NA 1:256 or greater) were selected for further evaluation. In trial III, piglets possessing high MDA levels were immunized with CSF-E2 subunit vaccine or LPC vaccine at 3 and 6 weeks of age. After a live attenuated vaccine was boosted at 16 weeks of age to mimic a mild to moderate CSFV infection in the field, the antibody level in CSF-E2 subunit vaccine immunized piglets was sharply converted at 20 weeks of age, revealing that an effective memory immune response was elicited by CSF-E2 subunit vaccine even under a high level of MDA pressure on early immunization (Fig. 4). All CSF-E2 subunit vaccine immunized pigs showed adequate protective immunity throughout the entire trial period to marketing. In contrast, more diverse and one in six LPC vaccine immunized piglets exhibited an extremely low antibody response (blocking percentage 6.15%) after the mimic boosted, indicating evidence of MDA interference on live attenuated vaccine even though pigs were immunized with two shots of live attenuated vaccine. Pigs with an insufficient antibody level might become a flaw in CSF prevention and create a leak in vaccination control in endemic pig farms.

One of the advantages of live attenuated CSF vaccines is that they may protect immunized pigs against CSFV infection as early as 5 days post-vaccination [16]. Hence, live attenuated CSF vaccines are believed to be suitable for emergency vaccination in outbreak herds [27]. Oral vaccination with a live attenuated vaccine (GPE-strain) was administrated in a wild boar population to control a reemerging CSF outbreak in domestic pig farms since September 2018 in Japan [39–41]. Nevertheless, according to the surveillance of domestic pig farms and wild boar population in March 2019, an extensive number of CSFV positive animals were detected in nearby areas [40]. However, it is still difficult to differentiate vaccinated pigs from infected animals, and prolonged surveillance should be followed after the execution of live attenuated vaccine [27]. The CSF-E2 subunit vaccine demonstrated high safety, the prevention of virus transmission, and non-interference by MDA, therefore, the combination of CSF-E2 subunit vaccine and live attenuated CSF vaccine (LPC vaccine) was used in trial IV. Piglets from CSF-E2 subunit vaccine immunized sows with high MDA level were immunized with CSF-E2 subunit vaccine at 3 weeks old, and another group was immunized with LPC vaccine at 12 and 16 weeks old to reduce MDA interference and to evaluate the immune response induced by different vaccination programs. All CSF-E2 subunit vaccine immunized piglets had high and adequate CSF-specific antibody levels during weaning to the finishing stage demonstrating that vaccine induced sufficient protective efficacy. Although the LPC vaccine immunized piglets had an adequate mean antibody level at four to 20 weeks of age, two out of ten pigs had a scanty antibody level at 12 and 16 weeks of age (Fig. 5). This result indicated the prospect of using the CSF-E2 subunit vaccine in sows to achieve consistent and long-lasting MDA that provides a strategy of late primary immunization with live attenuated CSF vaccine in piglets after 12 weeks of age. The combination of using the CSF-E2 subunit vaccine in sows and piglets or using the CSF-E2 subunit vaccine in sows followed by live attenuated CSF vaccine at a late stage might provide more flexibility in designing vaccination programs in field farms to prevent CSF. Moreover, sows immunized with CSF-E2 subunit vaccine at 3 and 5 weeks before parturition showed significantly lower CSFV RNA detecting ratio in saliva than sows with the long-term application of live attenuated CSF vaccine before insemination. Trial results identified that there was less risk of horizontal or vertical transmission risk and could provide another tool to conduct essential surveillance during reemerging outbreaks and revaccination procedures.

## Conclusions

In the present study, several animal trials, including an SPF pig model, MDA interference analysis, and combination strategies for immunization, were conducted to evaluate the efficacy of the CSF-E2 subunit vaccine in a conventional pig farm. The trial results may provide valuable information in the use of the CSF-E2 subunit vaccine to avoid the major drawbacks associated with live attenuated CSF vaccine and to increase vaccination efficiency in contribution to the approaches of CSF eradication.

## Supplementary Information

**Additional file 1: Figure S1.** Correlation between CSFV-specific antibody blocking percentage and NA titer. **Figure S2.** Gross and microscopic lesions of group C pigs. **Figure S3.** Detecting of CSFV infection in tissues.

## Data Availability

The datasets used and/or analyzed during the current study are available from the corresponding author on reasonable request.

## Abbreviations

CSF: Classical swine fever
SPF: Specific-pathogen-free
MDA: Maternal derived antibody
CSFV: Classical swine fever virus
OIE: World Organization for Animal Health
PRRSV: Porcine reproductive and respiratory virus
PCV2: Porcine circovirus type 2
NA: Neutralizing antibody
DPC: Days post-challenge
LPC: Lapinized Philippines Coronel strain
ABSL-2: Animal biosecurity level II
IACUC: Institutional Animal Care and Use Committee
TCID_50_: 50% tissue culture infective dose
EDTA: Ethylenediaminetetraacetic acid
Cp: Crossing point
CV: Coefficient of variation
