# Supplementary material for: Evaluation of classical swine fever E2 (CSF-E2) subunit vaccine efficacy in the prevention of virus transmission and impact of maternal derived antibody interference in field farm applications
Source: Porcine Health Manag. 2021 Jan 11;7:9. doi: 10.1186/s40813-020-00188-6 (PMC7798205; doi:10.1186/s40813-020-00188-6)
Supplement: Supplementary file 1 — Additional file 1: Figure S1. Correlation between CSFV-specific antibody blocking percentage and NA titer. Figure S2. Gross and microscopic lesions of group C pigs. Figure S3. Detecting of CSFV infection in tissues. [file 40813_2020_188_MOESM1_ESM.docx]

**Additional file 1**

Figure S1. Correlation between CSFV-specific antibody blocking percentage and neutralizing antibody (NA) titer. There were 162 serum samples (black dots) collected from field farms for serum surveillance after immunization of CSF-E2 subunit vaccine. All serum samples were analyzed for the NA titer and IDEXX CSF Ab test kit according to manufacturer’s instruction. The Pearson correlation coefficient analysis showed high correlation between the NA titer and blocking percentage (r = 0.94, p<0.001). The linear regression analysis (blue line) revealed significant agreement between two analysis results (R^2^ = 0.88, p<0.001). The blocking percentage greater than 40% may indicate sufficient NA titer (>1:32, dashed line) to protect individual animal and prevent virus transmission among pig population.


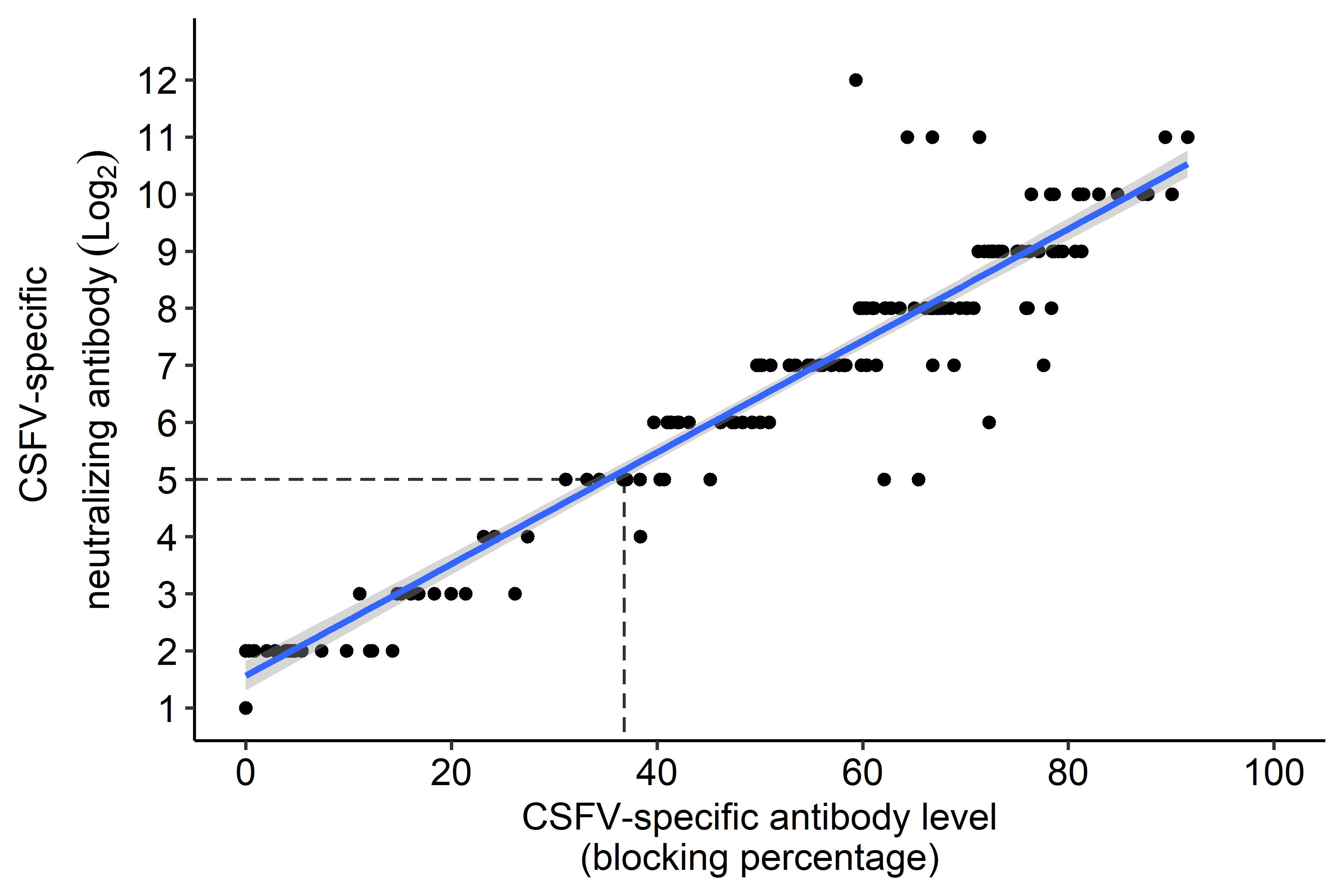


Figure S2. Gross and microscopic lesions of group C pigs. (a) Cyanosis on the tip of ears. (b) Cyanosis on the tip of the legs. (c) Multiple abscesses on the tonsils. (d) Kidney ecchymosis. (e) Ileocecal valve hemorrhage and ulceration. (f) Mesenteric lymph nodes hemorrhage and enlargement. (g) Disseminated intravascular coagulation (DIC) on the skin of the ear. (h) Tonsil abscesses. (i) Non-suppurative encephalitis, gliosis, cerebrum. (j) Interstitial nephritis, DIC and mononuclear inflammatory cell infiltration. (k) Spleen focal necrosis. (l) Lymph node, lymphoid depletion, and hemorrhage.

| (a) | (b) | (c) |
| --- | --- | --- |
| 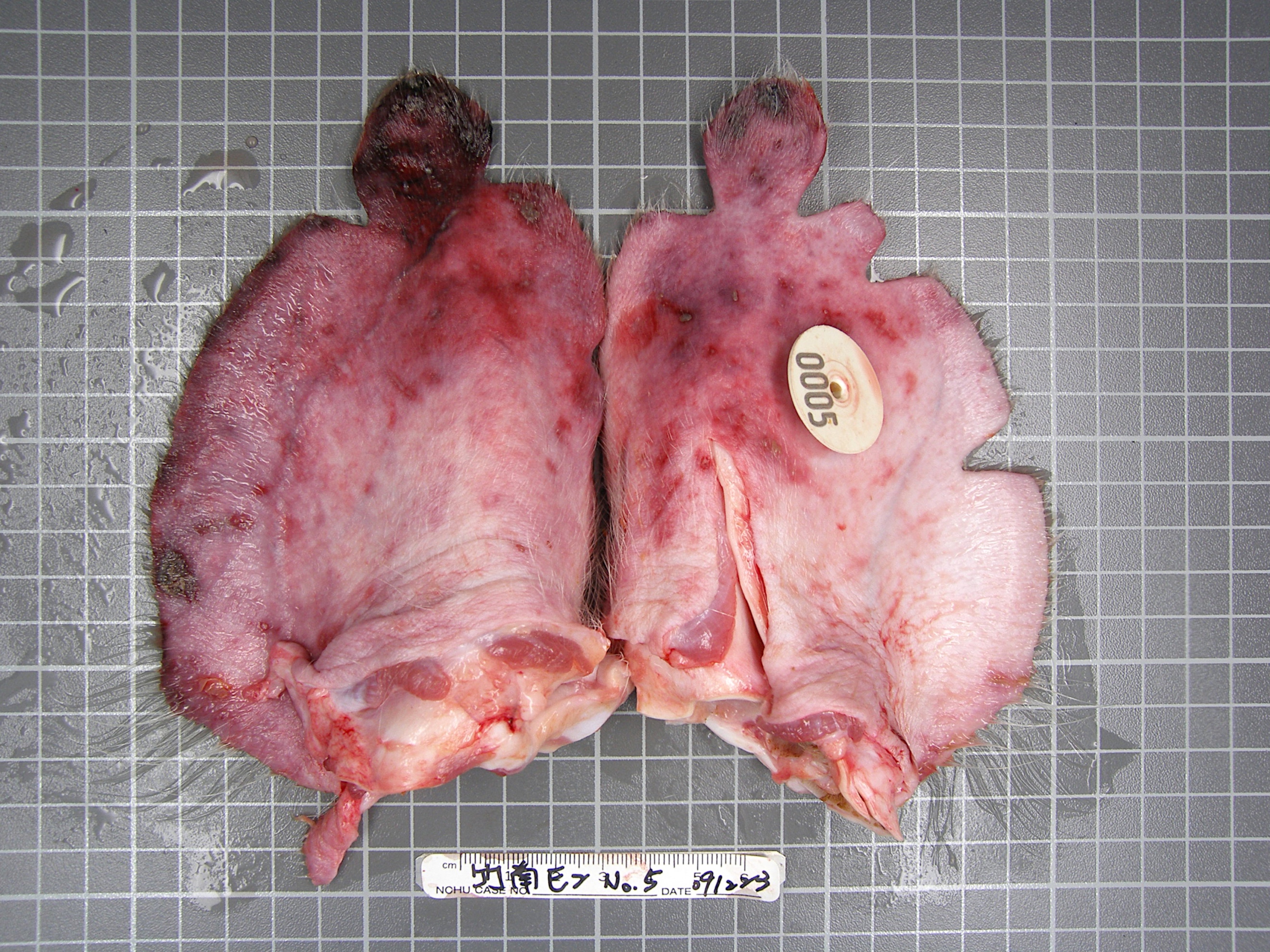 | 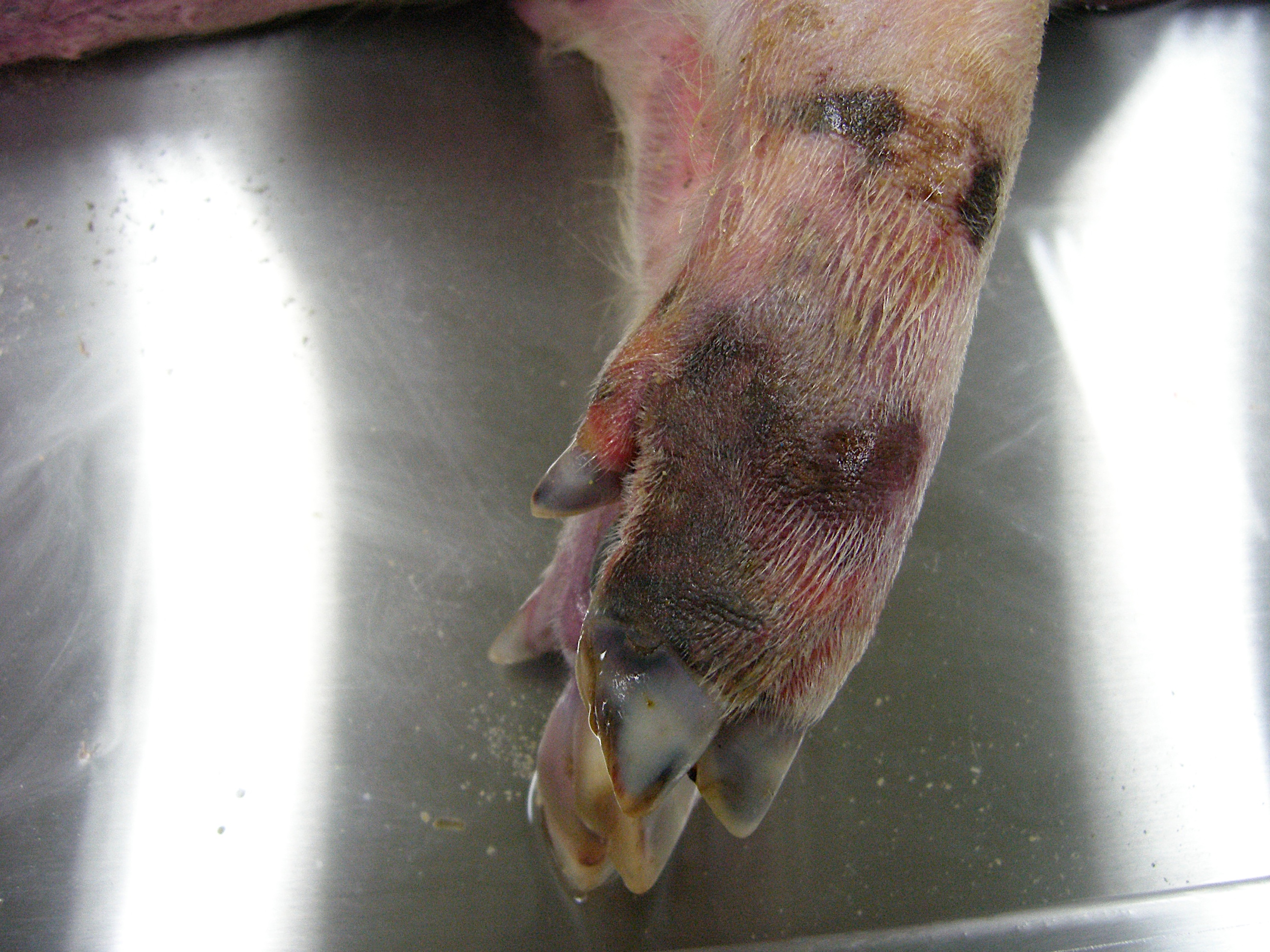 | 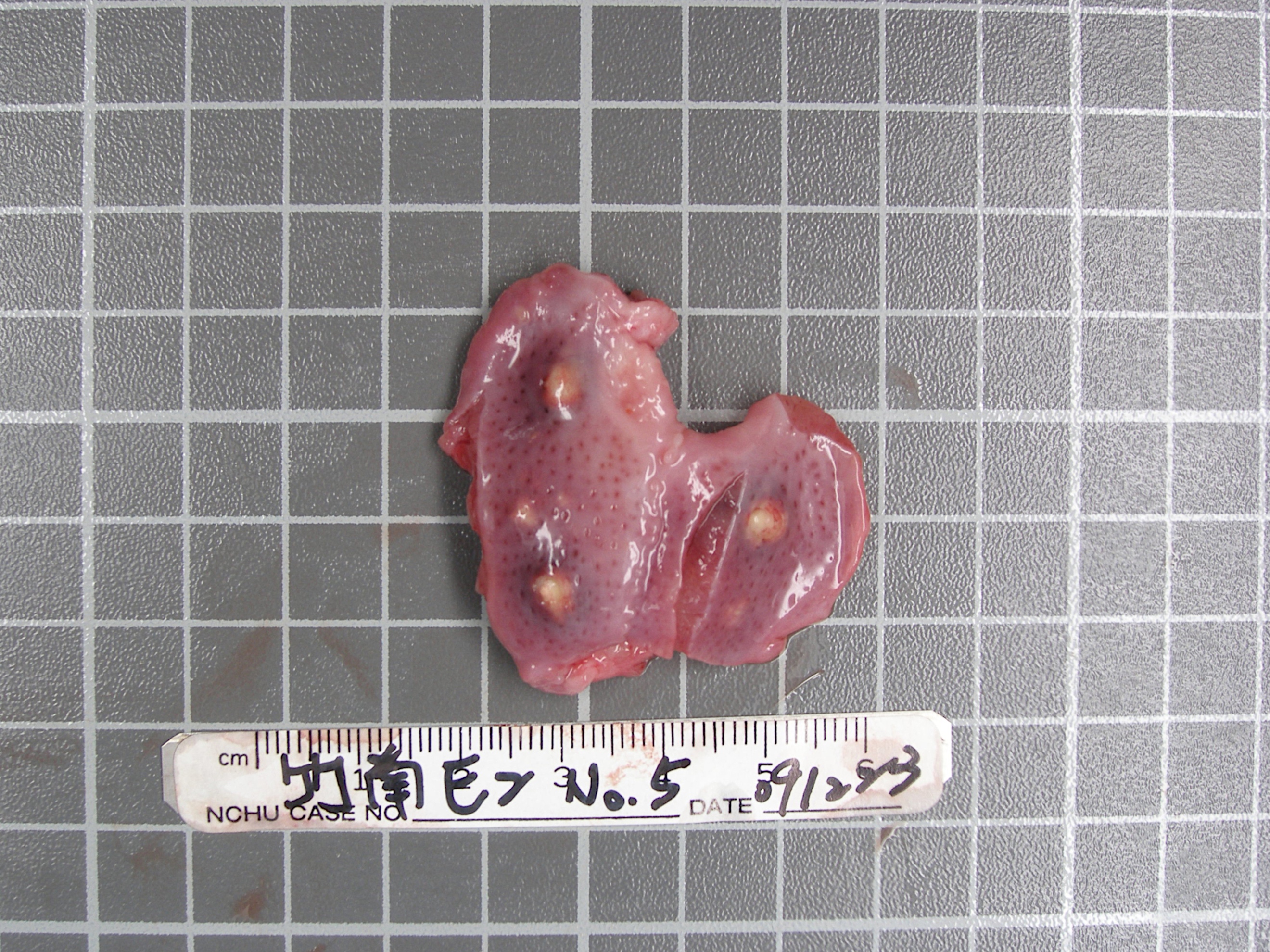 |
| (d) | (e) | (f) |
| 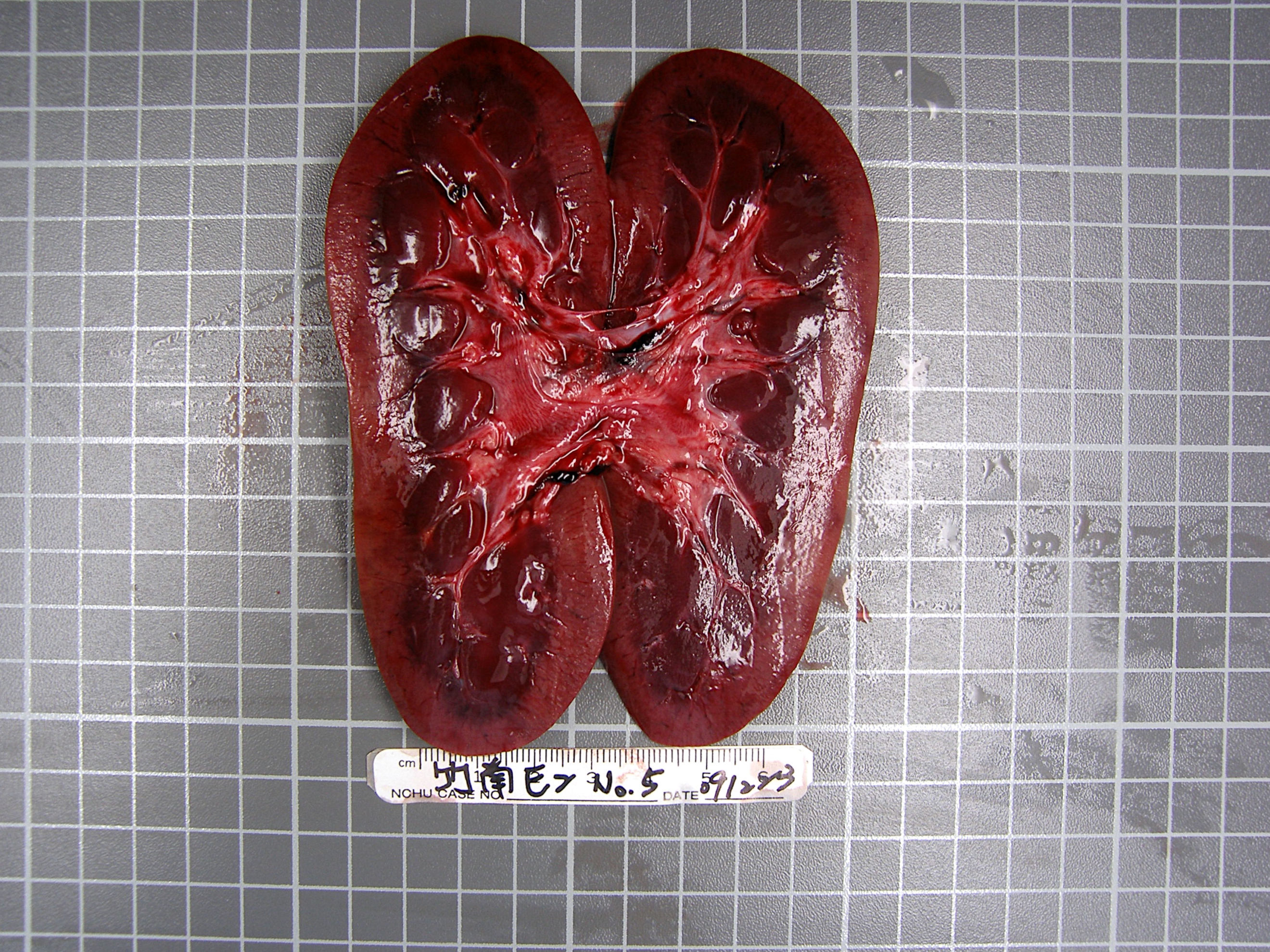 | 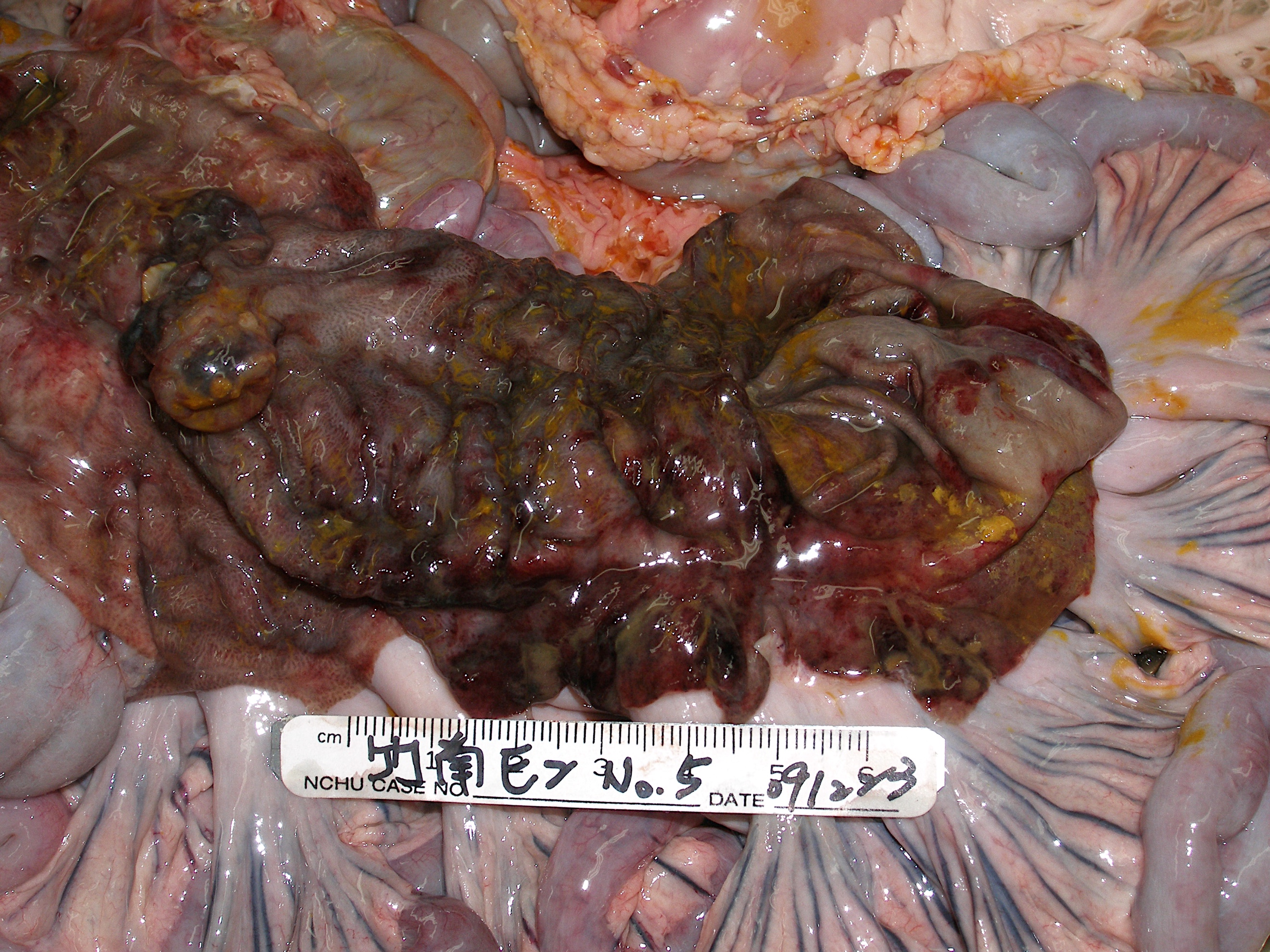 | 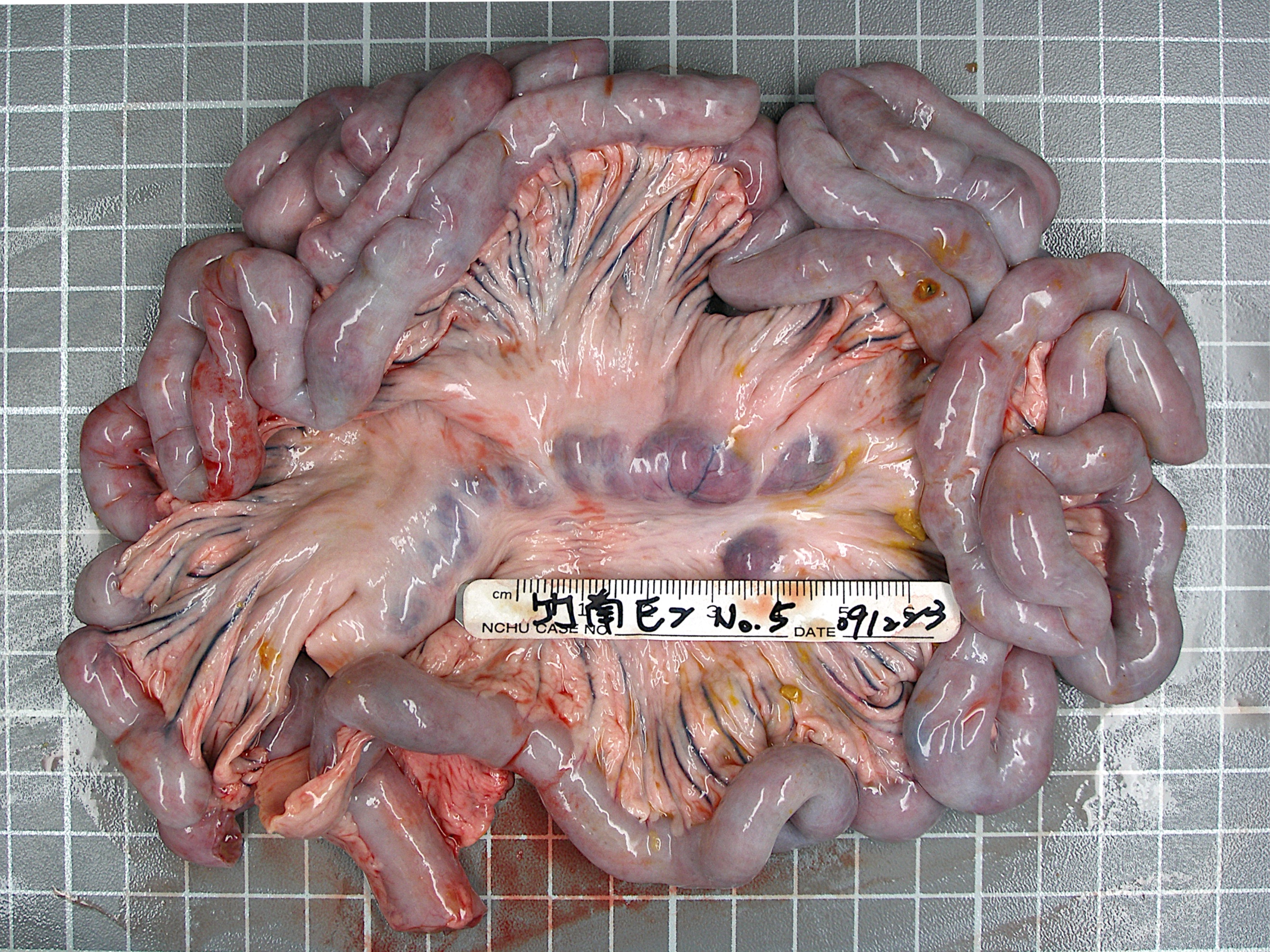 |
| (g) | (h) | (i) |
| 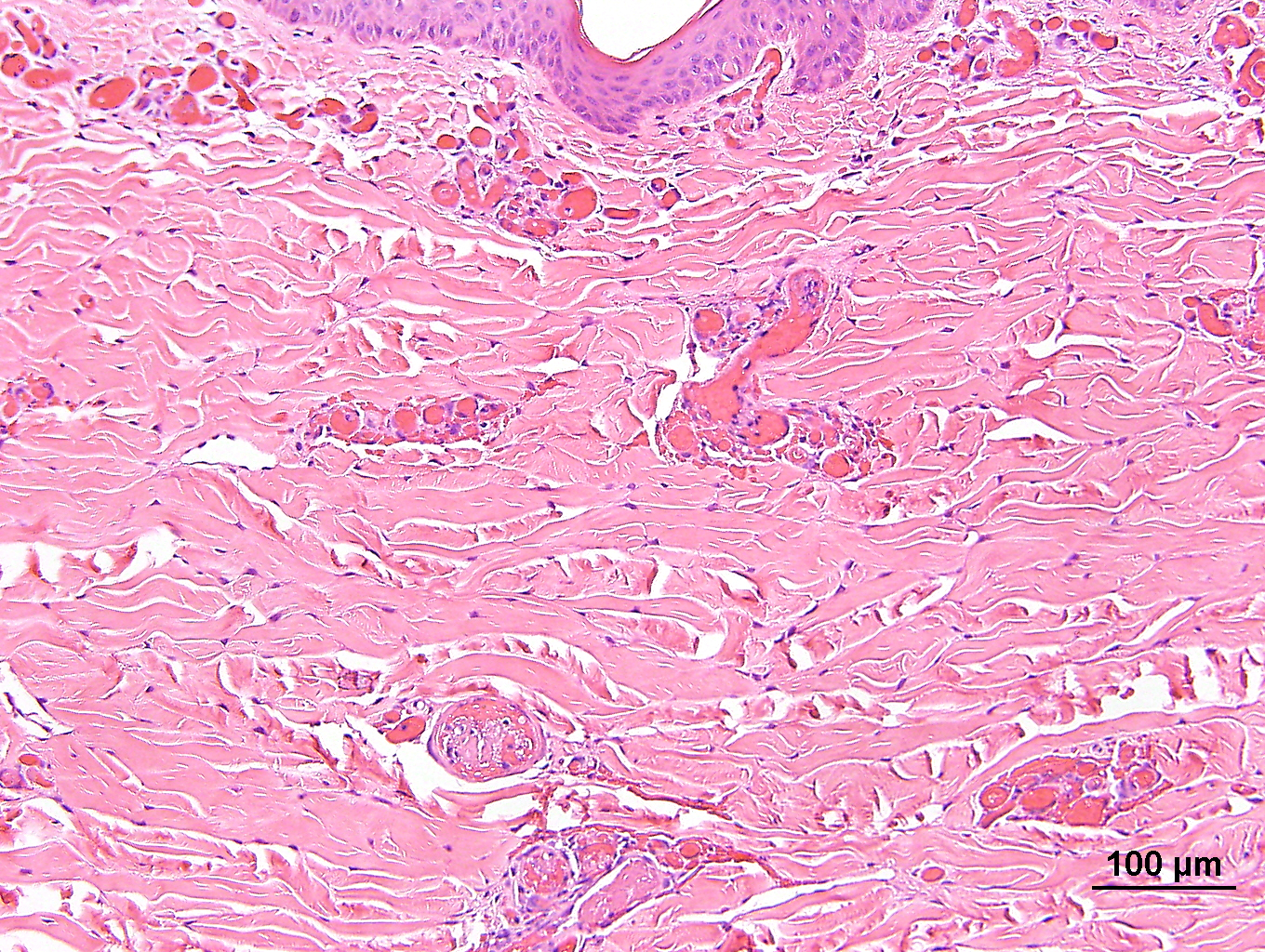 | 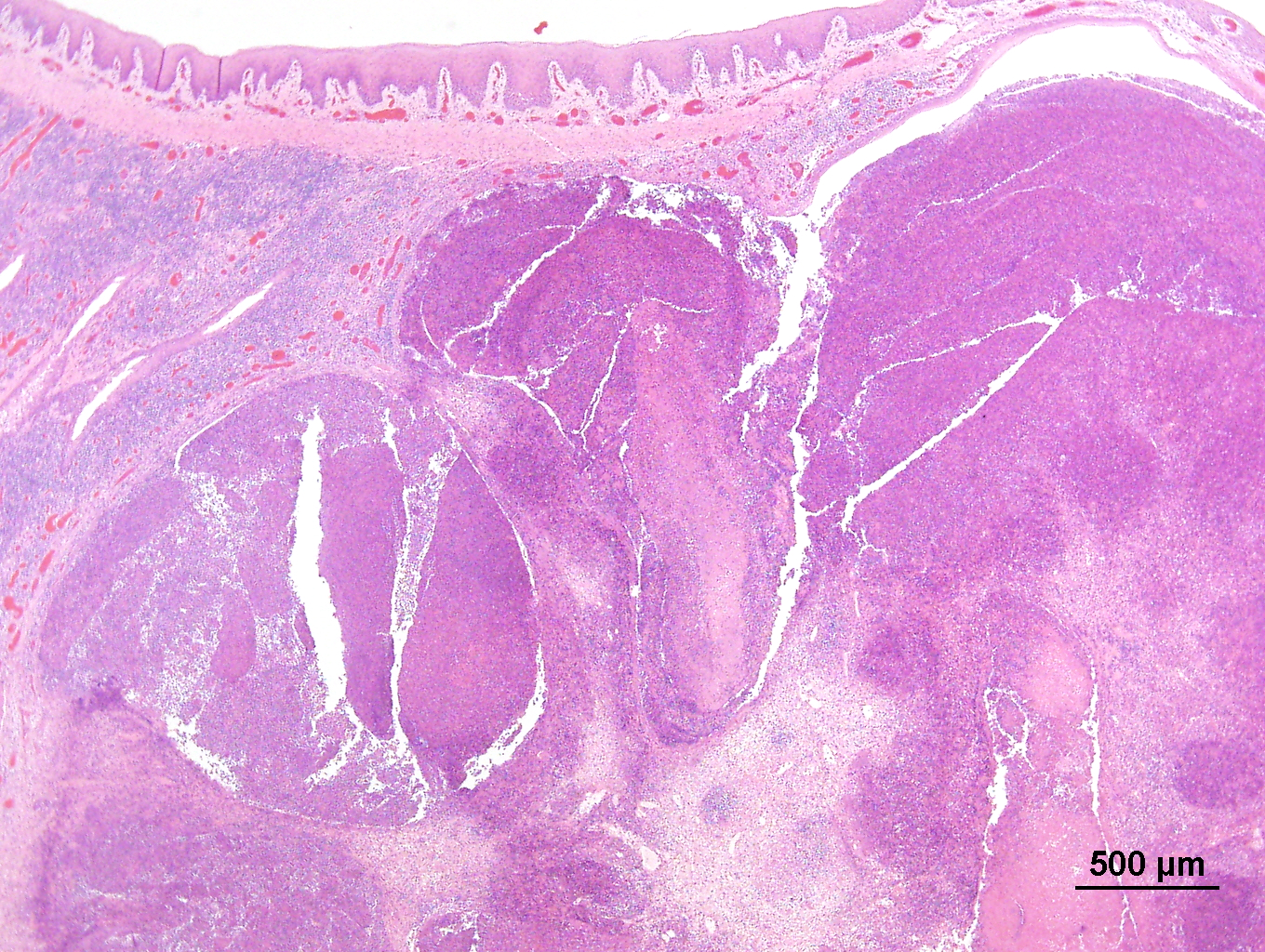 | 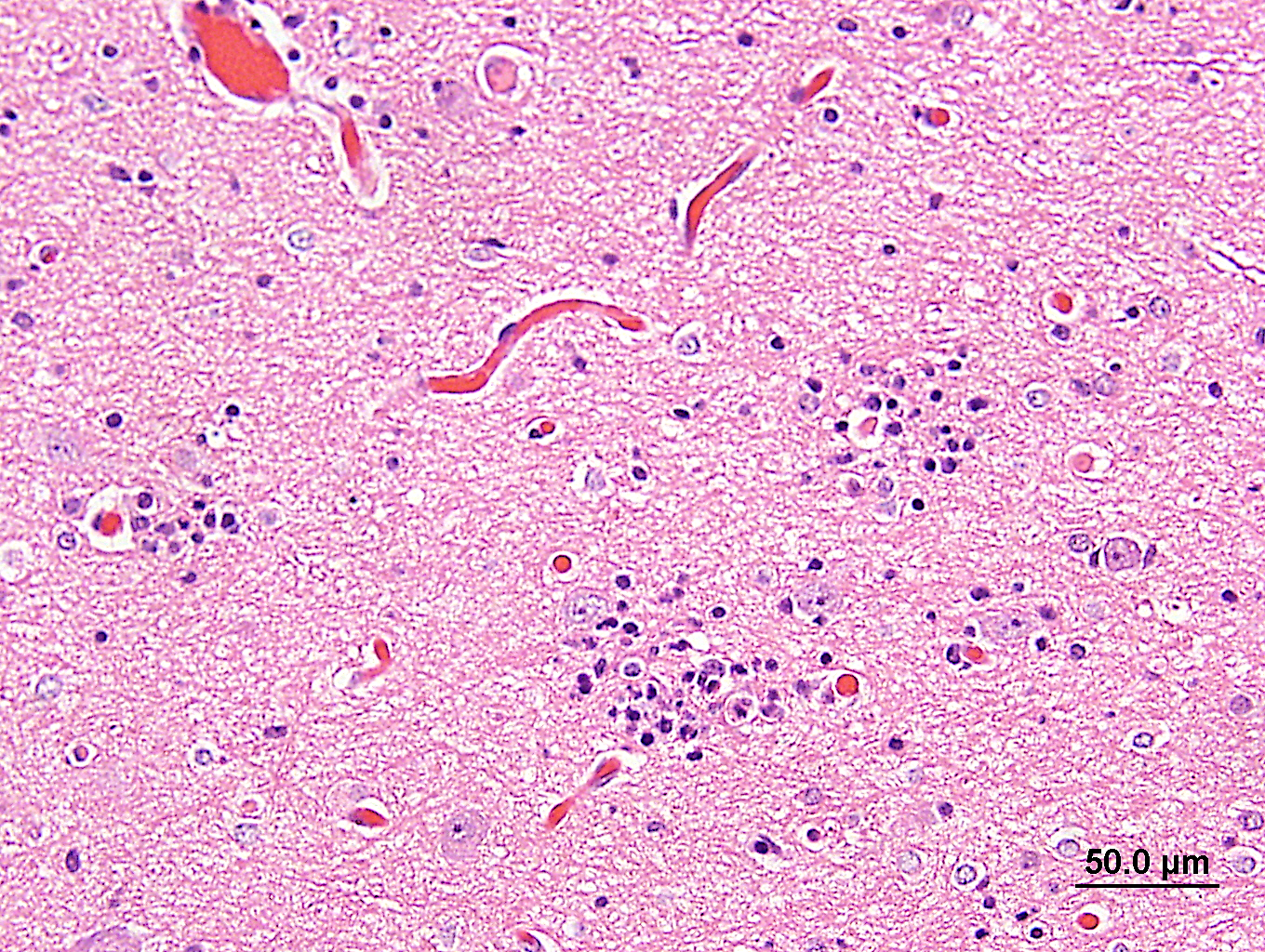 |
| (j) | (k) | (l) |
| 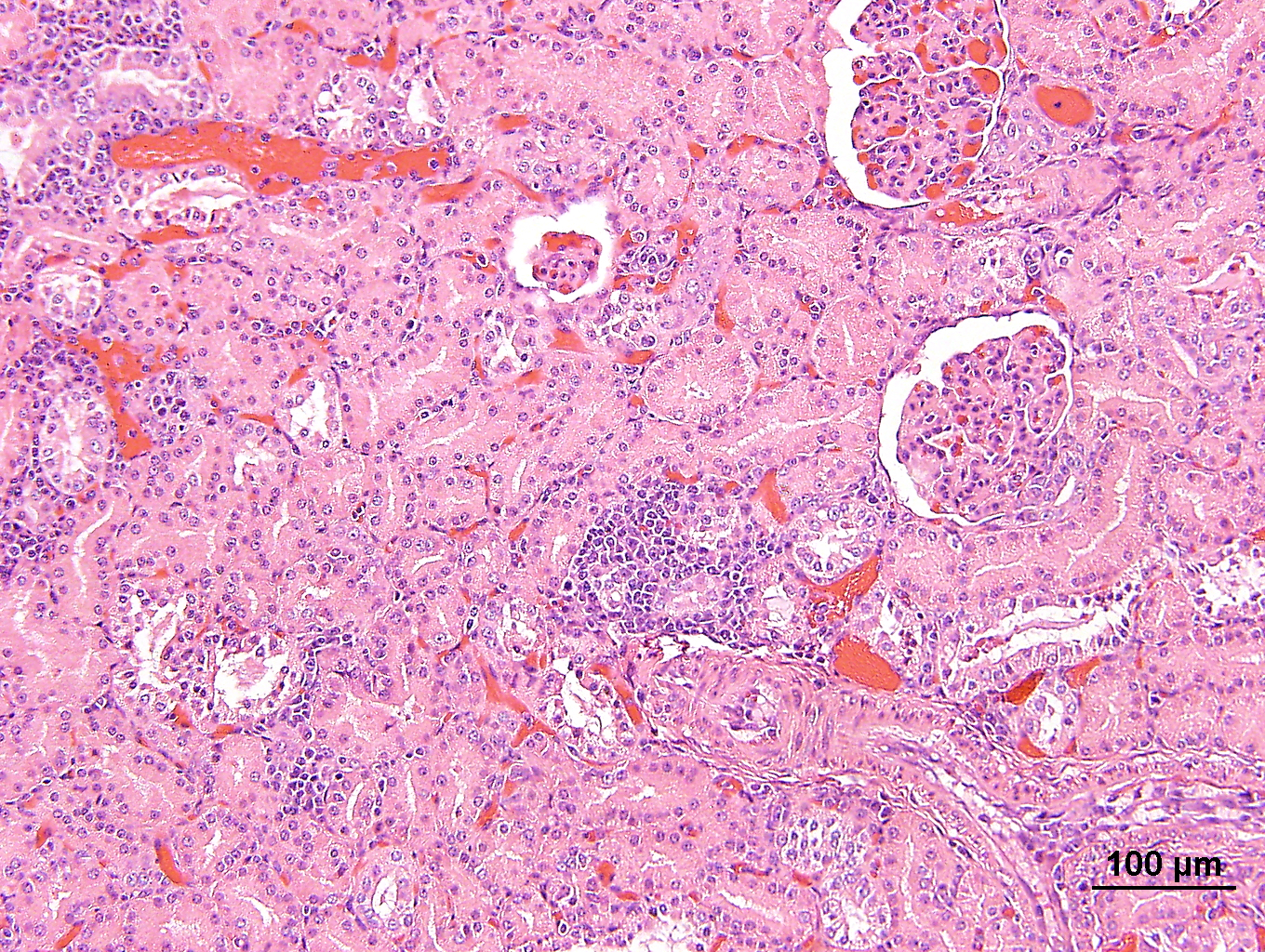 | 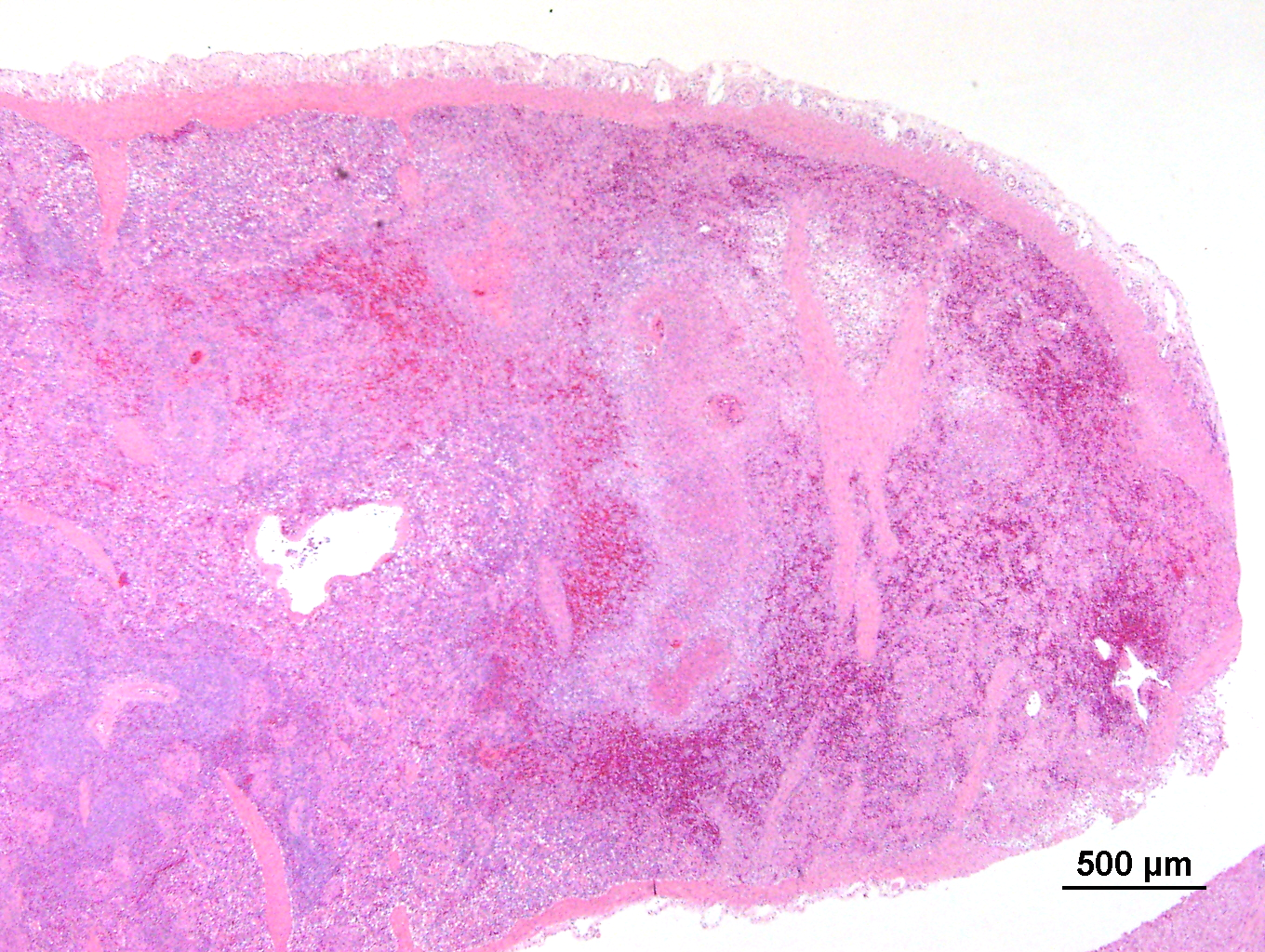 | 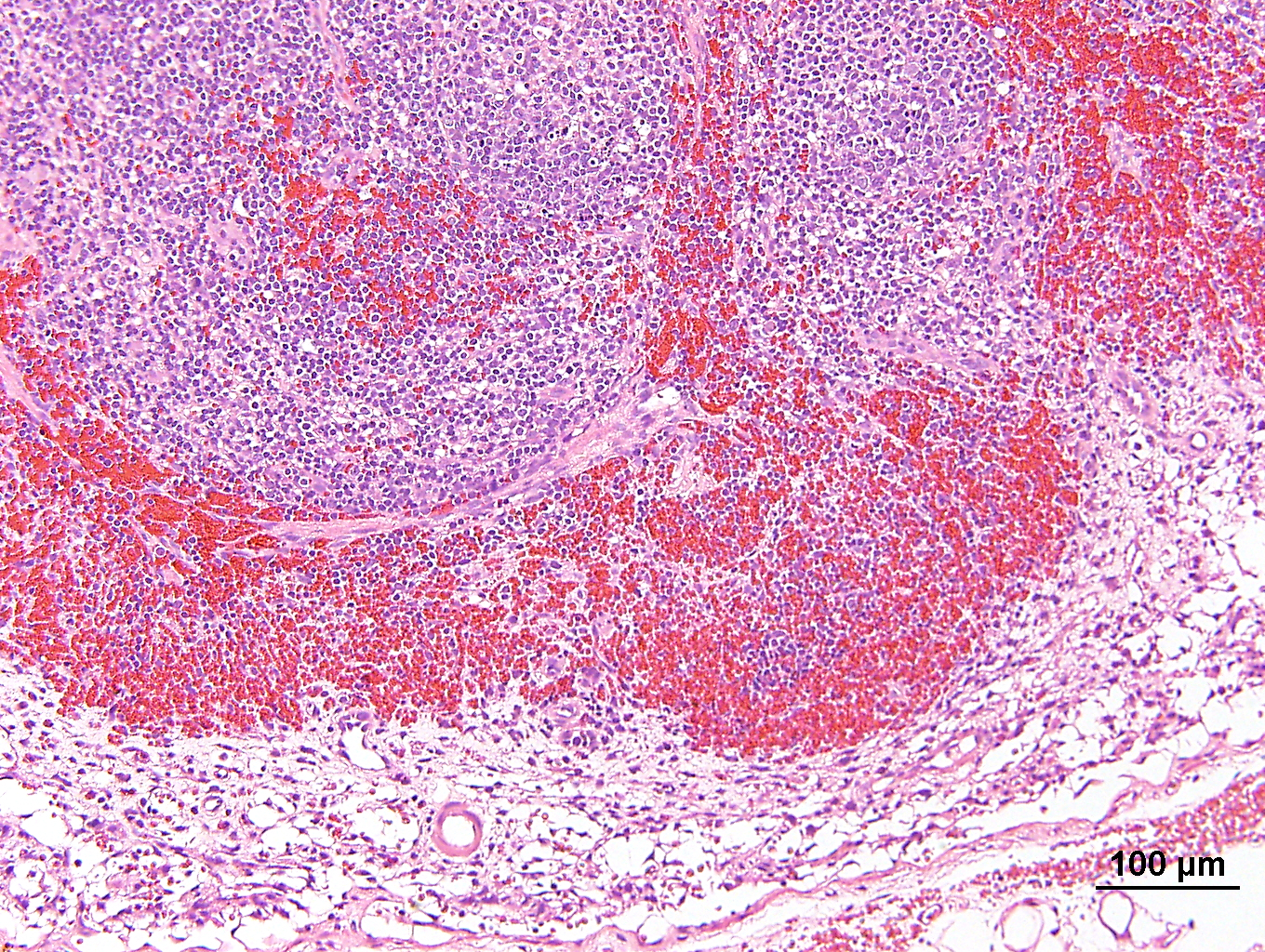 |

Figure S3. Detecting of CSFV infection in tissues. The CSFV antigen was detected using immunohistochemical staining with WH303 monoclonal antibody (PA0826, Veterinary Laboratories Agency, Weybridge, UK) (1:1000). The cells that were positive for viral antigen appear dark-brown. (a) Non-suppurative encephalitis, Cerebrum. (b) Interstitial pneumonia with DIC, Lung. (c) Hepatitis, mononuclear inflammatory cells infiltration at portal triad, Liver. (d) Lymph node with lymphoid depletion.

|  | H&E | IHC |
| --- | --- | --- |
| (a) | 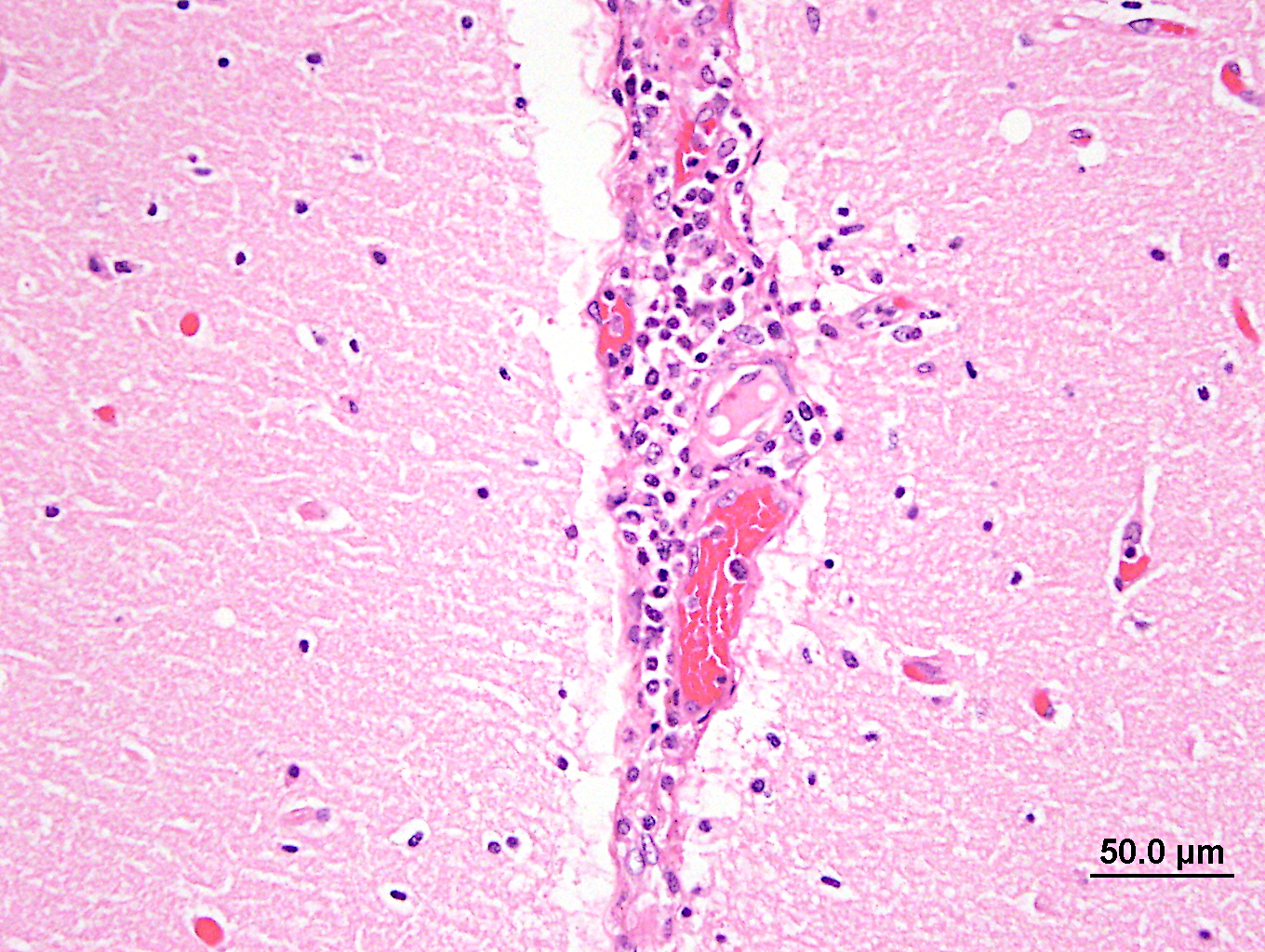 | 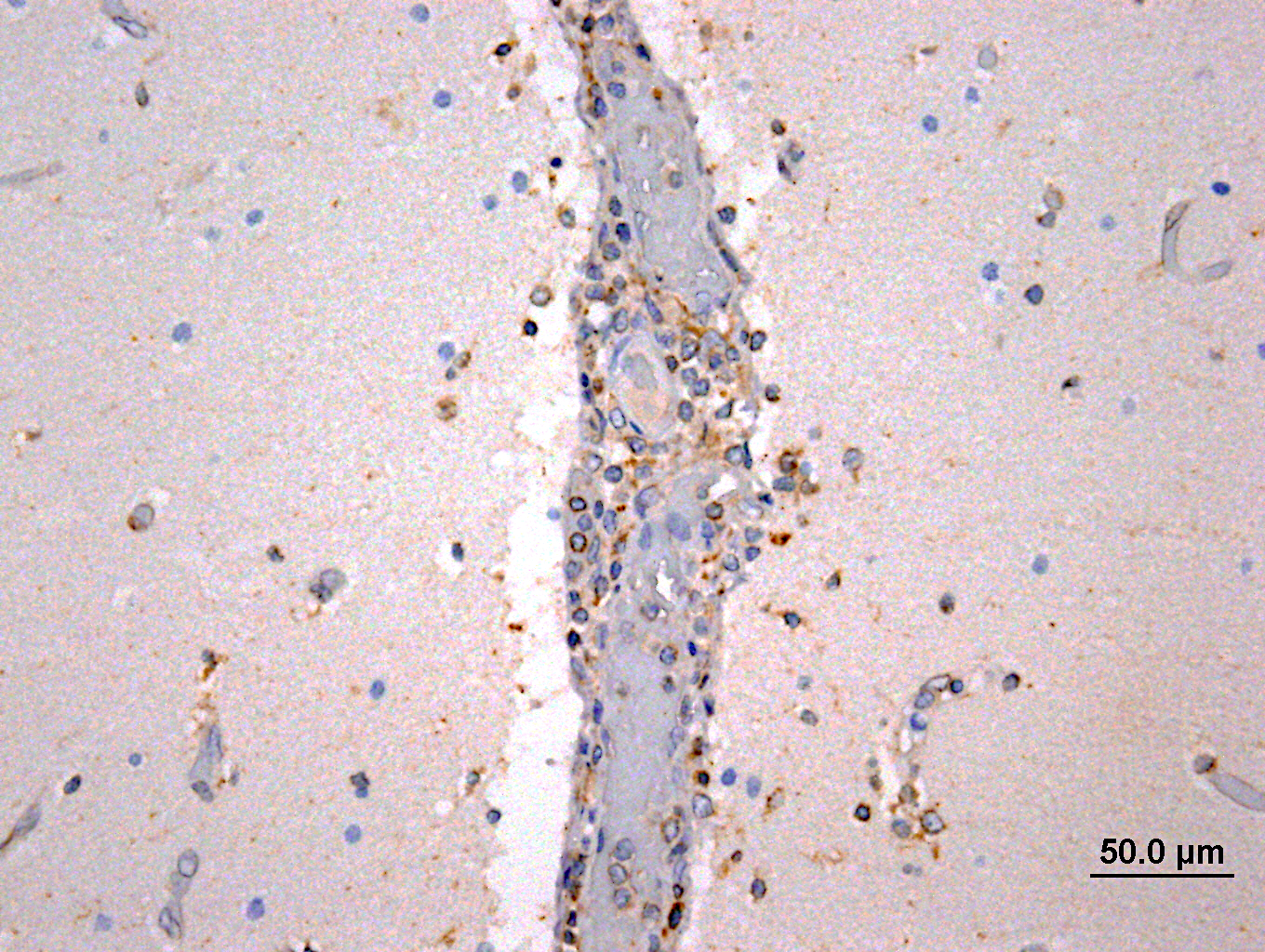 |
| (b) | 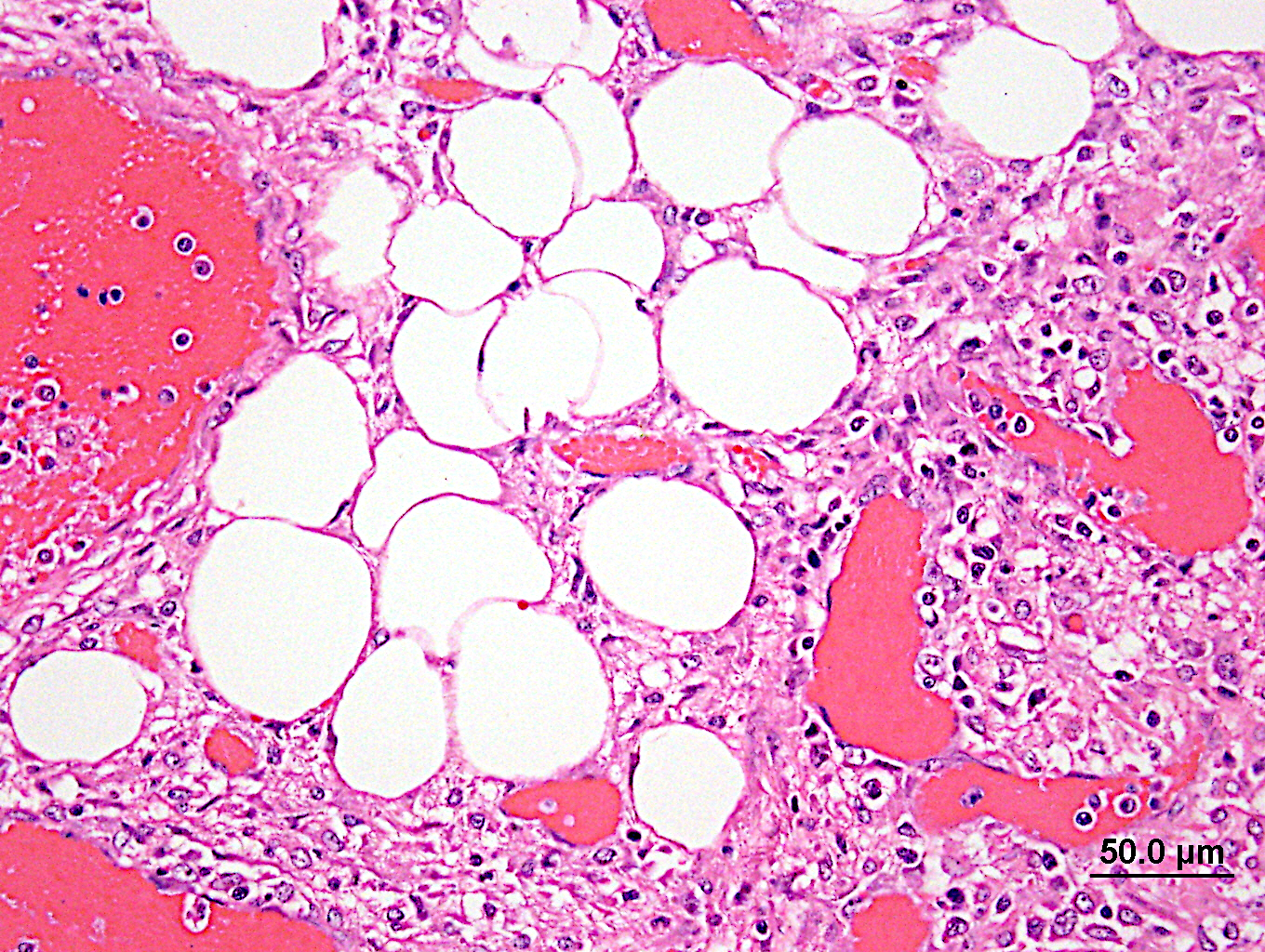 | 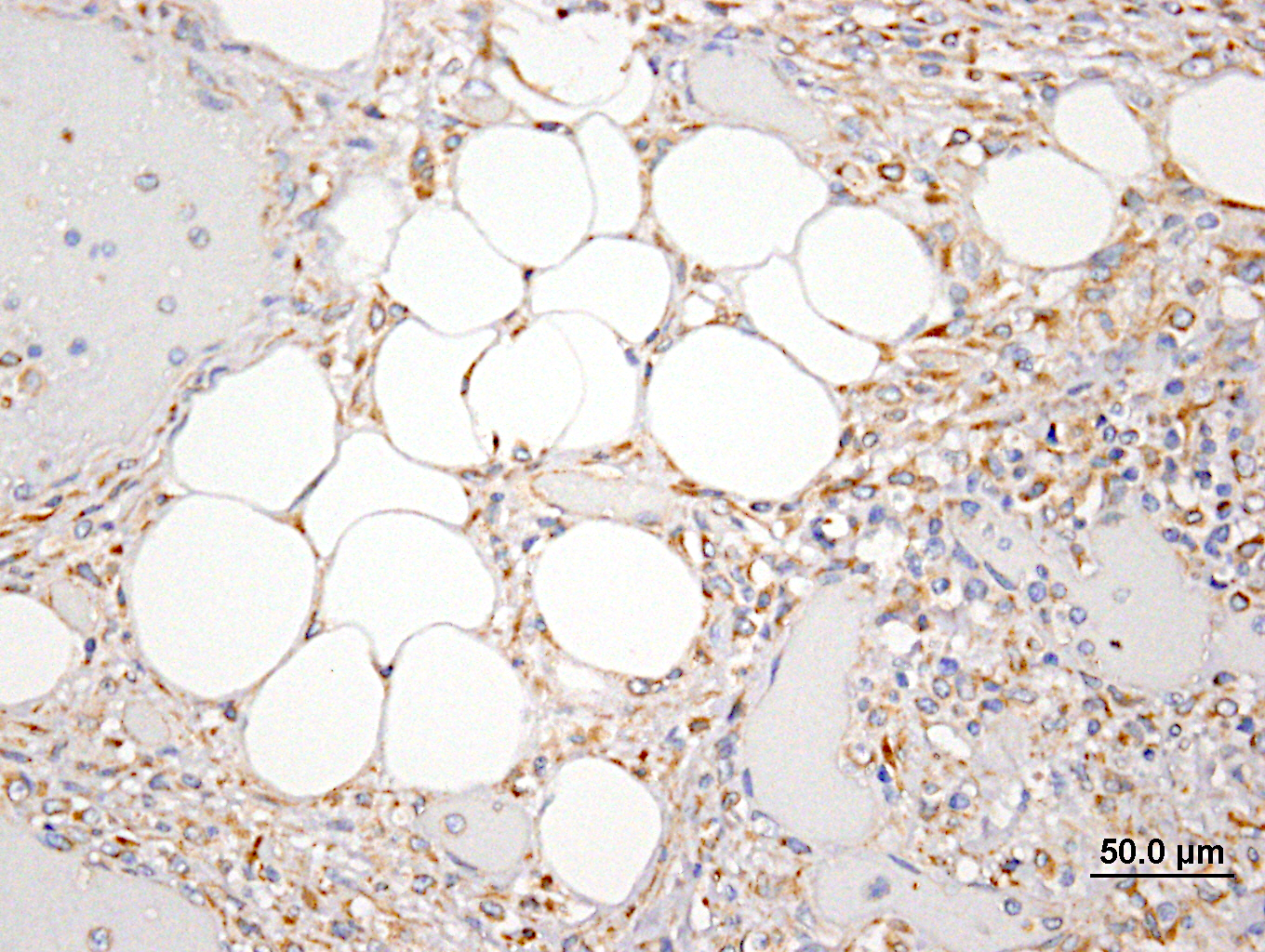 |
| (c) | 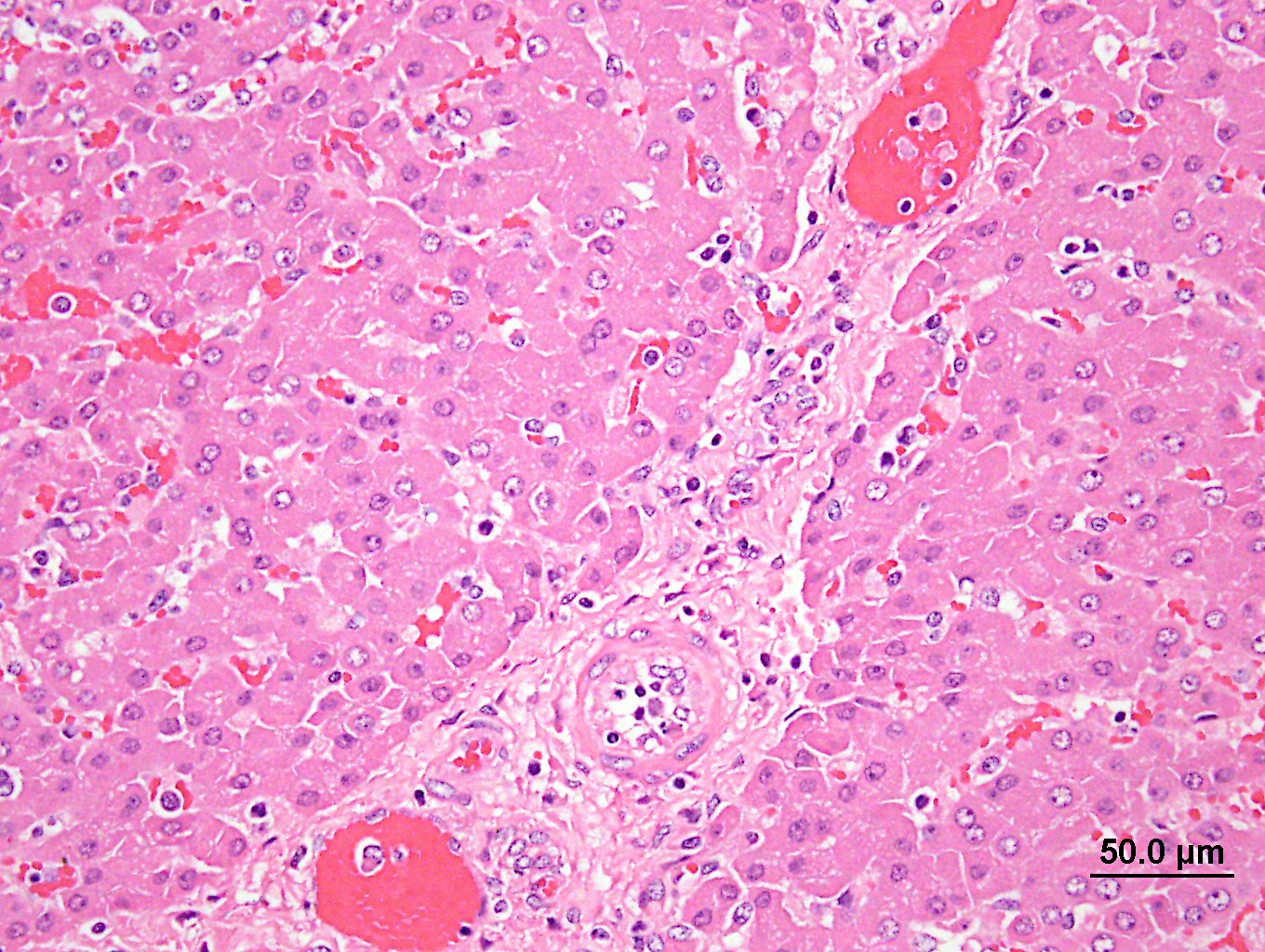 | 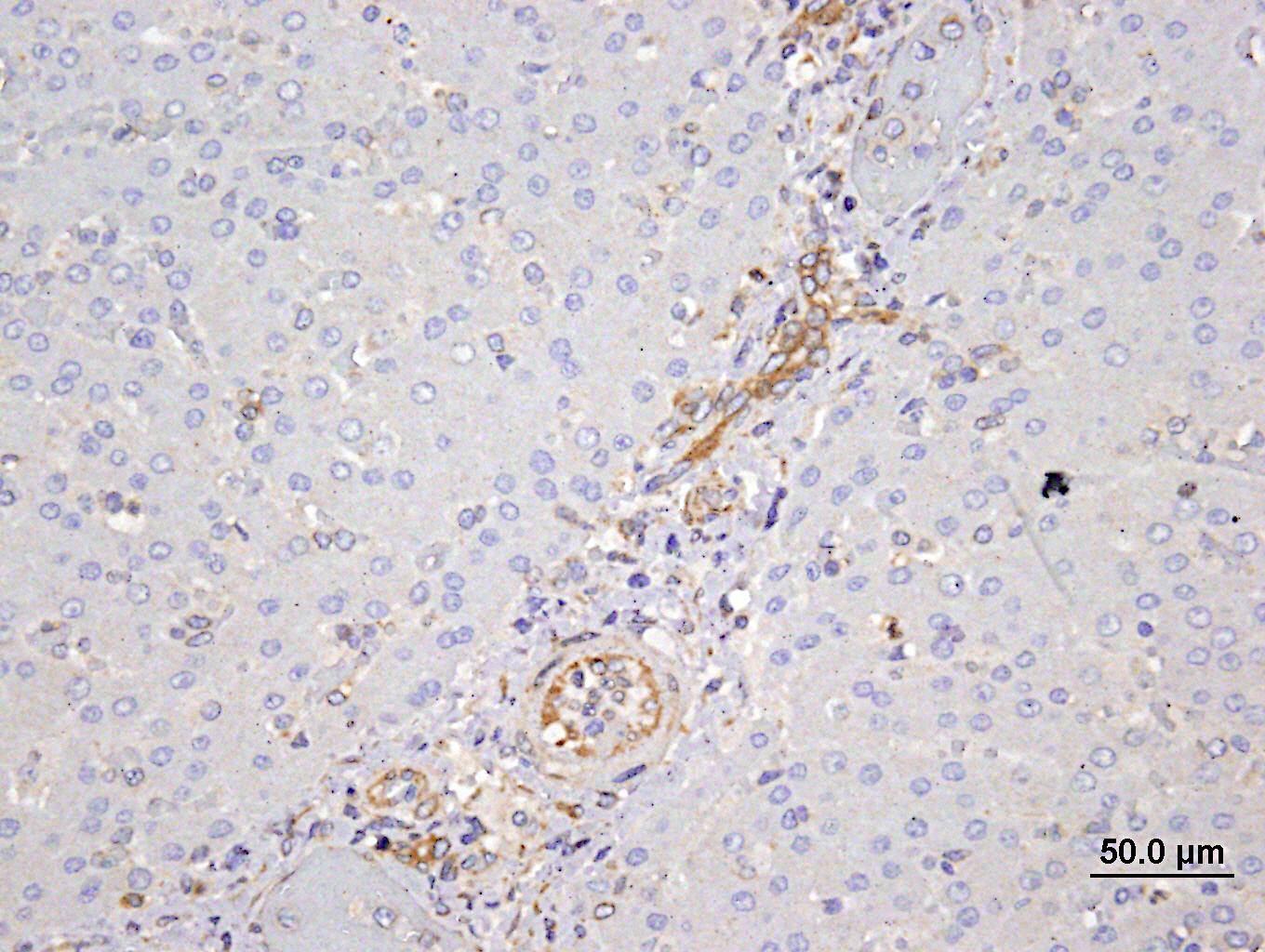 |
| (d) | 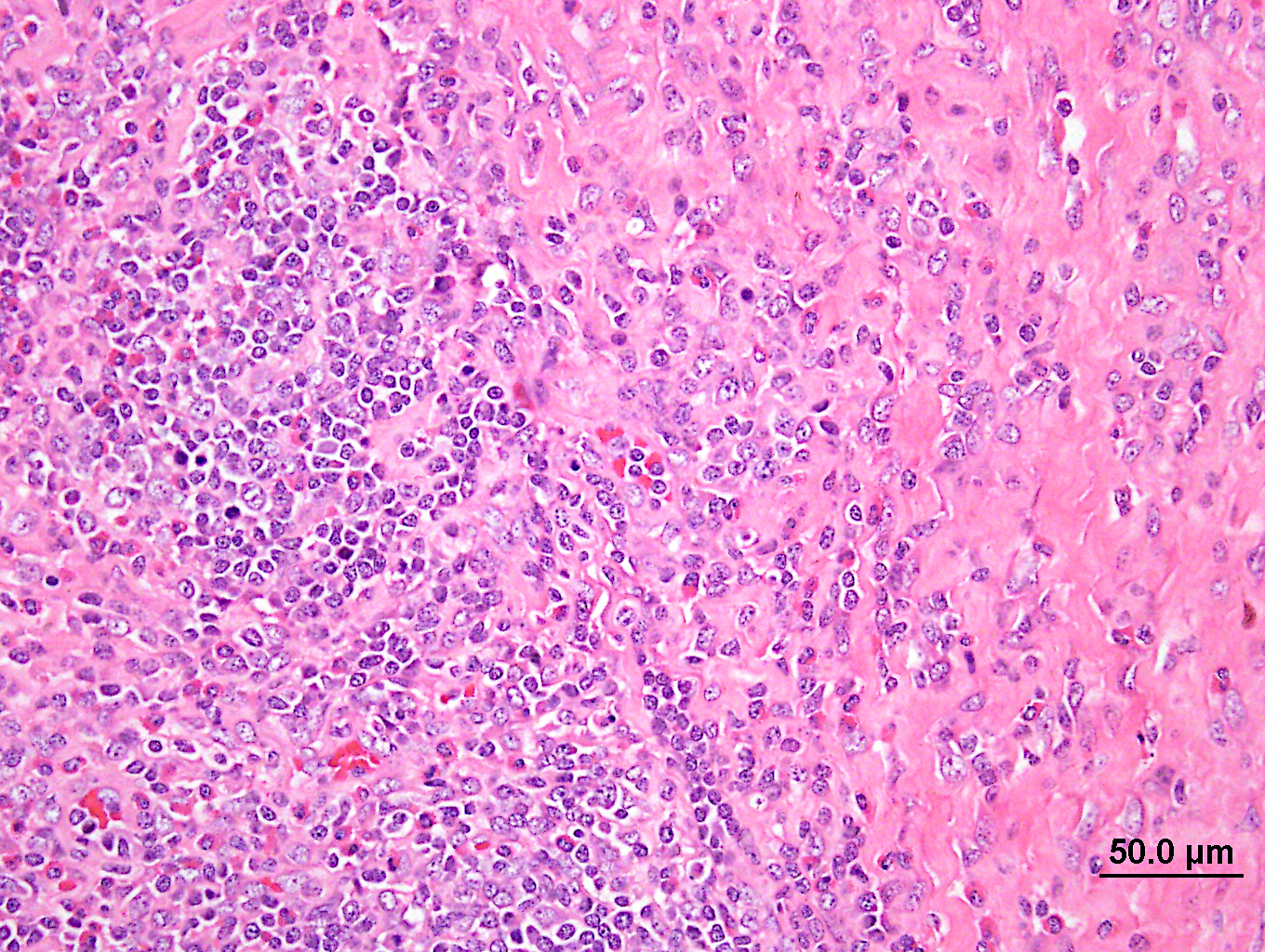 | 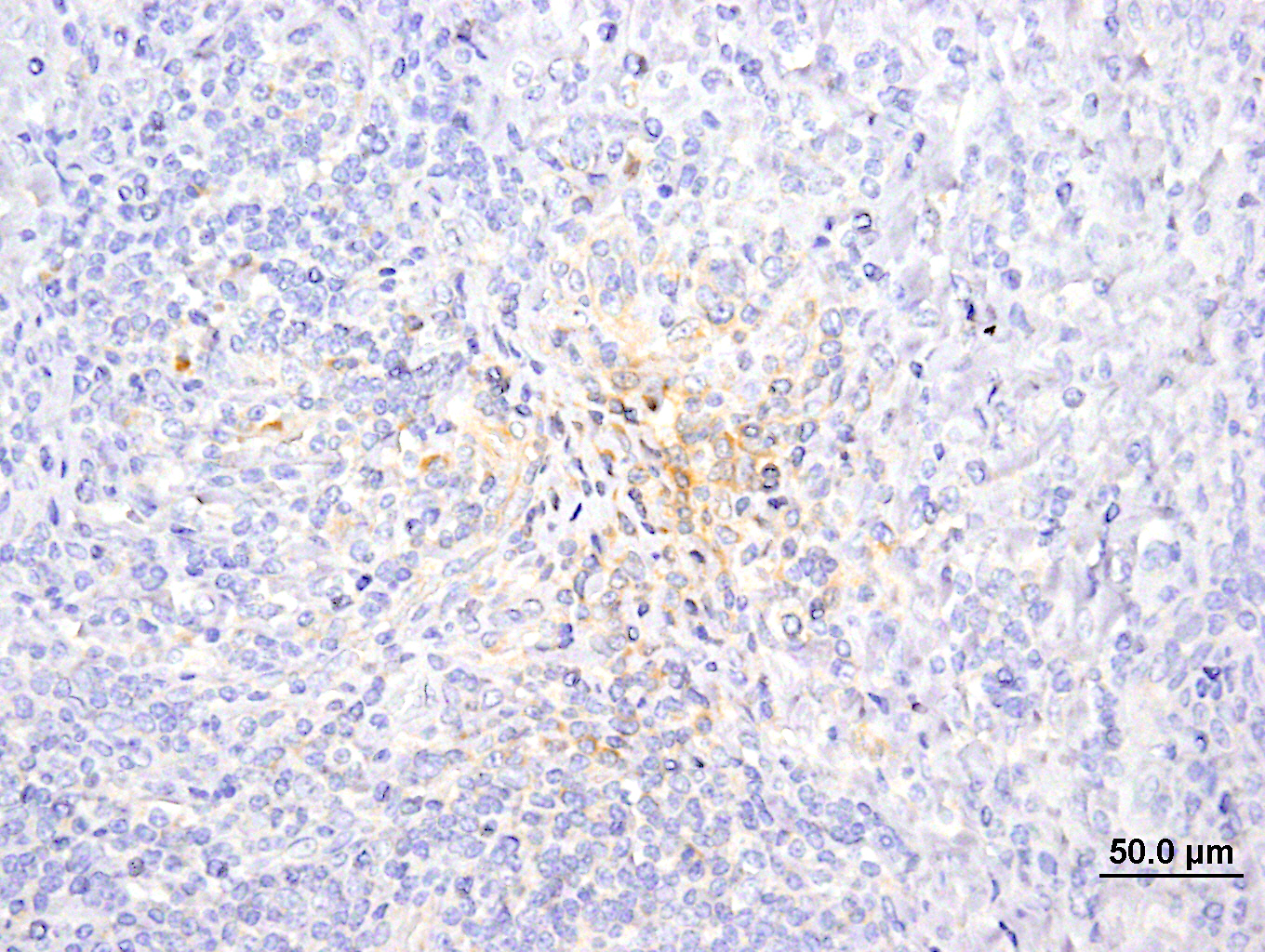 |
